# Phenotypic Antimicrobial Resistance Profiles and Provisional Epidemiological Cut-Off Values of *Edwardsiella anguillarum* Isolated from Farmed Nile Tilapia (*Oreochromis niloticus*) in Brazil, with Exploratory Data on *Edwardsiella tarda*

**DOI:** 10.3390/microorganisms14030523

**Published:** 2026-02-24

**Authors:** Natália Amoroso Ferrari, Vittória Cueva Segura da Silva, Pamela Giovana Turini, Julia Faria de Souza, Raffaella Menegueti Mainardi, Mayza Brandão da Silva, Alene Santos Souza, Gabriel Diogo Guimarães, Maisa Fabiana Menck-Costa, Marco Rozas-Serri, Mariene Miyoko Natori, Renata Galetti, Ulisses de Padua Pereira

**Affiliations:** 1 Laboratory of Fish Bacteriology, Department of Preventive Veterinary Medicine, State University of Londrina, Londrina 86057-970, Brazil; natalia.amoroso@uel.br (N.A.F.); vittoria.cuevas@uel.br (V.C.S.d.S.); pamelaturini@gmail.com (P.G.T.); juliafaria.souza@uel.br (J.F.d.S.); raffaellammveter@gmail.com (R.M.M.); brandao.mayza@gmail.com (M.B.d.S.); alenesantos47@gmail.com (A.S.S.); gdiogo705@gmail.com (G.D.G.); maisa.menckcosta@uel.br (M.F.M.-C.); 2 Pathovet Laboratory, Ribeirão Preto 14025-020, Brazil; marco.rozas@pathovet.cl (M.R.-S.); mariene.natori@pathovet.cl (M.M.N.); renata.galetti@pathovet.cl (R.G.)

**Keywords:** aquaculture, antimicrobial resistance, *Edwardsiella* spp., provisional local epidemiological cut-off value (pECV), multiple antibiotic resistance (MAR) index, One Health

## Abstract

Antimicrobial resistance in bacteria associated with aquaculture, such as *Edwardsiella* spp., represents an emerging challenge because of their relevance to fish health and their potential impact on animal, environmental, and human health. In this study, we primarily investigated the antimicrobial susceptibility profiles of *Edwardsiella anguillarum* isolated from farmed Nile tilapia (*Oreochromis niloticus*) in Brazil. Based on our findings, herein, we propose provisional local epidemiological cut-off values (pECVs) using the normalized resistance interpretation method, with data for *Edwardsiella tarda* included as an exploratory context. Fifty isolates (31 *E. anguillarum* and 19 *E. tarda*) collected between 2017 and 2025 were tested against 28 antibacterial agents using the disk diffusion method. Based on the pECVs, isolates were classified as wild type (WT) or non-WT (NWT), and the multiple antibiotic resistance (MAR) index was calculated. Most *E. anguillarum* isolates remained susceptible to several classes, although NWT and multidrug-resistant profiles were detected with a MAR index of 0.68, suggesting selective pressure in intensive tilapia farming systems. These findings support the use of local, species-specific pECVs for resistance surveillance in aquaculture, highlighting the importance of continuous antimicrobial resistance monitoring in aquaculture from a One Health perspective.

## 1. Introduction

Edwardsiellosis is a septicemic disease that affects a wide range of aquatic hosts, resulting in substantial economic losses and posing a threat to aquaculture systems worldwide [1]. The disease is caused by species of the genus *Edwardsiella*, four of which affect fish: *E. tarda*, *E. anguillarum*, *E. ictaluri*, and *E. piscicida* [2]. *Edwardsiella tarda* is most frequently associated with clinical conditions in fish, whereas *E. anguillarum* has been described as an emerging and highly virulent pathogen, particularly affecting tilapia from tropical regions [2,3,4]. Outbreaks have been associated with high fish mortality rates, reduced growth performance, increased treatment costs, and trade losses, collectively resulting in substantial financial impacts in regions with intensive production. In an outbreak in oscar fish (*Astronotus ocellatus*), an ornamental species, *E. tarda* has been reported to cause 100% mortality within 240 h [5]. In another outbreak affecting tilapia in Korea, a strain of *E. anguillarum* has been reported to cause significant mortality and economic loss [6]. Recent studies have indicated that infections caused by *E. anguillarum* can lead to acute septicemia and rapid mortality, reinforcing its increasing relevance in warm-water aquaculture [7,8]. Taxonomic revisions have direct implications for antimicrobial susceptibility studies because historical data may reflect heterogeneous species assignments.

These bacteria are gram-negative bacilli that, in addition to compromising animal health and productivity, hold potential public health relevance. *Edwardsiella tarda* has been linked to human infections, including bacteremia and septicemia, which are often associated with the consumption of raw fish or environmental exposure to infections [9]. The zoonotic potential of *E. tarda*, combined with its widespread distribution in the intestinal microbiota of fish and other aquatic organisms [1], highlights the need for integrated surveillance strategies from a One Health perspective [5], particularly in regions where raw or minimally processed fish are commonly consumed [6,10]. In addition, there is limited knowledge regarding the zoonotic potential of *E. anguillarum* and the molecules used for its treatment.

In this context, the use of antimicrobials remains a main measure for controlling bacterial infections in fish, including those caused by *Edwardsiella* spp. [1,10,11]. However, the pressure exerted by these drugs favors the selection of drug- or multidrug-resistant (MDR) strains [1,12]. Furthermore, the indiscriminate or prolonged use of these agents accelerates and intensifies this phenomenon, transcending aquaculture and posing a global public health threat under the One Health Framework [13]. Moreover, *Edwardsiella* spp. play an important role in the dissemination of resistance genes, often carried by mobile genetic elements, such as plasmids [1]. Classical genes, such as *blaTEM* (β-lactamase gene) and *tetA* (tetracycline resistance gene), have been reported in *E. tarda*, reinforcing its function as a reservoir and vector of resistance to other microorganisms [3,10]. In several countries, only a few molecules have been validated and authorized for use in fish, such as in Brazil, where only oxytetracycline and florfenicol have been officially approved [14]. Based on routine diagnostic cases in Brazil (unpublished data), reduced therapeutic responses to these compounds have been occasionally observed in infections caused by *Edwardsiella* spp., a scenario also suggested by in vitro findings reported in the literature [10].

Additionally, species within the genus *Edwardsiella* exhibit differences in ecological distribution and host predilection [1]. For example, *E. anguillarum* has been isolated from Nile tilapia and other species, such as seabream, eels, and milkfish [4,7], whereas *E. tarda* has an even broader host range, including freshwater and marine fish [10,15,16]. These species-specific associations suggest that distinct lineages circulate in different ecological niches, potentially impacting their antimicrobial resistance profiles.

Despite the growing number of reports on edwardsiellosis, significant methodological limitations remain. There are no official interpretive criteria, recommended by the CLSI (Clinical and Laboratory Standards Institute)/ EUCAST (European Committee on Antimicrobial Susceptibility Testing), specific to *Edwardsiella* spp., and the absence of standardized epidemiological cut-off values (ECVs) complicates the detection of early resistance and comparisons across studies [10]. Furthermore, although some studies have generated ECVs or susceptibility data for specific *Edwardsiella* spp., direct comparative evaluations of *E. tarda* and *E. anguillarum*, two species with increasing relevance in tropical aquaculture, are scarce [10]. This gap is particularly relevant because these species exhibit differences in their host preferences and pathogenicity profiles in cultured fish [7] and in the One Health context.

Considering the limited availability of isolates from each species in most routine aquaculture diagnostics, including in the present study, generating large datasets suitable for formal ECV determination is often not feasible. Thus, preliminary datasets are valuable as they provide the first comparative signals needed to guide future surveillance, refine hypotheses, and support the gradual construction of robust reference values.

Therefore, continuous monitoring of antimicrobial susceptibility in fish-associated bacteria is essential for elucidating the emergence and spread of resistance. In this scenario, even preliminary comparative data may make meaningful contributions by revealing the early differences between bacterial pathogen species.

In this study, we aimed to characterize the antimicrobial susceptibility profiles of *E. anguillarum* isolated from Nile tilapia and to propose provisional local ECVs (pECVs) using phenotypic data. The results obtained for *E. tarda* are presented as exploratory and contextual information rather than for a formal interspecies comparison.

## 2. Materials and Methods

### 2.1. Origin and Selection of Isolates

The *Edwardsiella* spp. isolates analyzed in this study were obtained from farmed Nile tilapia and submitted to the Fish Bacteriology Laboratory of the State University of Londrina (LABBEP–UEL) for microbiological diagnosis between 2017 and 2025.

After necropsy, the eye, brain, kidney, liver, and spleen fragments were aseptically collected and inoculated onto Mueller–Hinton blood agar (MHBA), which contains Mueller–Hinton agar (MHA; Himedia, Mumbai, India) supplemented with 5% defibrinated sheep blood. The plates were incubated at 29 ± 1 °C for 24–48 h for bacterial isolation. Initially, the colonies were evaluated for morphology and Gram staining, in addition to biochemical tests for genus-level identification.

As it is not possible to differentiate *Edwardsiella* species using only phenotypic tests, DNA was extracted using the DNeasy Blood and Tissue Kit (QIAGEN, Hilden, Germany), following the manufacturer’s instructions, for the molecular identification of species using multiplex polymerase chain reaction, as proposed by Da Costa et al. [17]. After identification, the isolates were preserved at −80 °C in a solution containing brain–heart infusion broth (Himedia, Mumbai, India) and 20% glycerol.

In this study, 19 isolates of *E. tarda* and 31 of *E. anguillarum* were collected from Nile tilapia (*Oreochromis niloticus*) originating from six Brazilian states, as shown in Table 1.

### 2.2. Antimicrobial Susceptibility Tests

The selected isolates were subcultured from frozen stocks onto MHBA and subjected to the disk diffusion method on MHA following the recommendations of the CLSI guideline VET03 for bacteria isolated from aquatic animals [18]. After a 24 h incubation at 28 °C, a bacterial inoculum was prepared and standardized to 0.5 McFarland (approximately 1.5 × 10^8^ CFU [colony-forming units]/mL) and subsequently seeded onto MHA plates. A total of 28 antibacterial agents were evaluated (piperacillin + tazobactam, aztreonam, meropenem, ceftazidime, amoxicillin, sulfazotrim, norfloxacin, tobramycin, ampicillin, cefazolin, imipenem, levofloxacin, cefoxitin, amoxicillin + clavulanic acid, ceftiofur, cefepime, ceftriaxone, cefotaxime, cefuroxime, florfenicol, amikacin, gentamicin, cephalexin, ciprofloxacin, tetracycline, enrofloxacin, streptomycin, and marbofloxacin). The antimicrobial panel was primarily composed of agents recommended for the susceptibility testing of *Enterobacterales*, ensuring coverage of the main antimicrobial classes relevant to this group. In addition, antimicrobial agents routinely used in the diagnostic workflow for fish bacterial isolates at the LABBEP–UEL were included because of their relevance in treating bacterial diseases in fish.

The inhibition zone diameters were measured in millimeters. The phenotypic production of extended-spectrum β-lactamases (ESBLs) and ampicillin cephalosporinase β-lactamases (ACBLs) was also assessed. For this purpose, antibiotic discs were strategically arranged on MHA plates to facilitate the detection of the characteristic inhibition patterns associated with these resistance mechanisms. *Escherichia coli* ATCC 25922 was included as the reference strain to monitor assay performance and verify disk viability. Moreover, internal laboratory controls previously characterized as positive were used to validate the phenotypic identification of ESBL and ACBL production.

### 2.3. Determination of ECVs and Multiple Antibiotic Resistance (MAR) Index

Considering that the CLSI and BrCAST (Brazilian Committee on Antimicrobial Susceptibility Testing)/ EUCAST interpretive criteria extrapolate values that have not been validated for fish pathogens, the isolates were not classified using these systems.

Instead, pECVs were determined using the normalized resistance interpretation (NRI) method available at https://www.bioscand.se/nri/ (accessed on 13 October 2025). Based on the pECVs, isolates were classified as wild type (WT) or non-WT (NWT) [19]. Considering the sample size, the pECVs calculated for *E. anguillarum* (*n* = 31) are presented as local and provisional reference values, whereas those estimated for *E. tarda* (*n* = 19) are presented as preliminary and exploratory. According to the authors’ recommendations, when disc diffusion datasets include fewer than 50 observations obtained at 28 °C, the standard deviation (SD) upper limit recommended for assays performed at 22 °C should be used for the analysis. As our dataset met these criteria (<50 observations at 28 °C), we adopted the corresponding threshold and considered an upper SD limit of <6.49 mm. NWT isolates were interpreted as resistant.

For each isolate, the MAR index was calculated as the ratio of the number of antibacterial agents to which the strain was resistant (NWT) to the total number of antibacterial agents used. The MAR index is a tool used to indicate the level of antibiotic pressure in a given source, with a threshold of 0.2 commonly used to identify environments at a high risk of antibiotic exposure [20].

For *E. tarda*, no pECV was established for tetracycline, owing to numerous small inhibition zones that prevented analysis; thus, 27 antibacterial agents were considered for the MAR index calculation. Isolates exhibiting resistance to three or more classes of antibacterial agents were classified as MDR [21].

### 2.4. Data Analyses

The results were compiled in Microsoft Excel and analyzed using RStudio v.2025.05.1 + 513. The English language of the manuscript was reviewed and polished using the AI language model ChatGPT (version GPT-5.2, OpenAI) to enhance clarity, grammar, and overall readability.

## 3. Results

The results are presented based on antimicrobial class to facilitate the interpretation of phenotypic susceptibility patterns between species. Phenotypic antimicrobial susceptibility testing revealed heterogeneous inhibition zone diameters among *E. tarda* (*n* = 19) and *E. anguillarum* (*n* = 31) isolates obtained from farmed Nile tilapia (*O. niloticus*) (Table 2 and Table 3; Supplementary Figures S1–S28). The quality control strain *Escherichia coli* ATCC 25922 yielded inhibition zone diameters within the expected reference ranges for all antimicrobial agents tested, validating the disk diffusion assays (Supplementary Table S1). The internal quality controls used for the phenotypic screening of ESBL and ACBL production also performed as expected, confirming the technical reliability of these procedures (Supplementary Figure S29).

Based on the pECV classification, both species showed high proportions of WT isolates for several antimicrobial classes. In the piperacillin + tazobactam treatment, 95% of *E. tarda* and 81% of *E. anguillarum* isolates were classified as WT. Third- and fourth-generation cephalosporins also exhibited high WT proportions, including ceftazidime (95% WT in *E. tarda* and 94% WT in *E. anguillarum*), cefepime (100% and 94% WT, respectively), and cefotaxime (89% and 81% WT) (Table 2).

Carbapenems showed high WT proportions in both species. In the imipenem treatment, 89 and 100% of *E. tarda* and *E. anguillarum* isolates were classified as WT, respectively, whereas in the meropenem treatment, 95 and 90% of the corresponding isolates were classified as WT, respectively. Aminoglycosides also showed predominantly WT profiles, particularly tobramycin (100% WT in *E. tarda* and 97% WT in *E. anguillarum*), amikacin (95% and 100% WT, respectively), and gentamicin (84% and 100% WT, respectively).

In contrast, an increased proportion of NWT isolates was observed for several antimicrobial agents. Among penicillins, in the amoxicillin and ampicillin treatments, 42% of *E. tarda* isolates were classified as NWT for each compound, whereas 3% of *E. anguillarum* isolates were classified as NWT. Amoxicillin and clavulanic acid elevated NWT proportions in both species, reaching 68% in *E. tarda* and 58% in *E. anguillarum*. When grouped by antimicrobial class, penicillins and fluoroquinolones presented higher NWT proportions than those of carbapenems, aminoglycosides, and advanced-generation cephalosporins.

For cephalosporins, *E. anguillarum* exhibited higher pECVs for ceftazidime, cefotaxime, cefuroxime, and cefazolin than did *E. tarda*. Ceftriaxone and cephalexin exhibited high WT proportions in *E. tarda* (89 and 95%, respectively), whereas in the cephalexin treatment, 48% of *E. anguillarum* isolates were classified as NWT.

Fluoroquinolone susceptibility profiles differed between the species. For *E. tarda*, norfloxacin and levofloxacin classified 89% and 58% of the isolates as NWT, respectively, whereas ciprofloxacin and marbofloxacin classified 95% and 100% of the isolates as WT, respectively (Table 3). In *E. anguillarum*, NWT proportions were observed for ciprofloxacin (42%), enrofloxacin (29%), norfloxacin (23%), and marbofloxacin (16%), whereas 90% of the isolates were classified as WT for levofloxacin.

Florfenicol classified 53% of *E. tarda* and 52% of *E. anguillarum* isolates as WT. Provisional pECVs for tetracycline could not be established for *E. tarda*, whereas *E. anguillarum* showed a distribution that allowed the classification of 52% of the isolates as WT and 48% as NWT.

Multidrug resistance, defined as resistance to three or more antimicrobial classes based on the pECV classification, was detected in 15 of the 19 *E. tarda* isolates (78.95%) and 22 of the 31 *E. anguillarum* isolates (70.97%). Non-MDR profiles were observed in 4 *E. tarda* isolates (21.05%) and 9 *E. anguillarum* isolates (29.03%).

**Figure 1:**
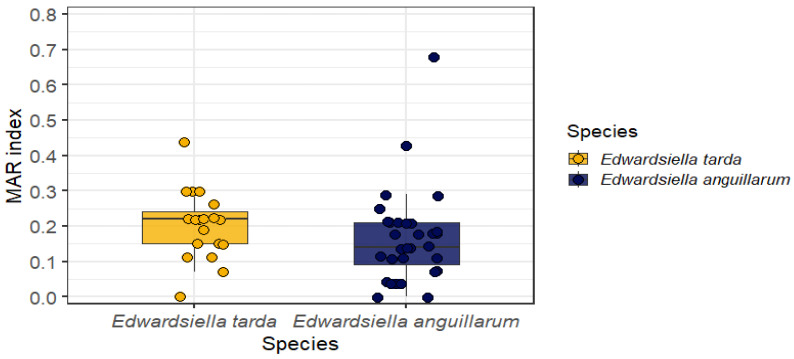
Multiple antibiotic resistance (MAR) index in *Edwardsiella* isolates. Distribution and individual variability of the MAR index between *Edwardsiella tarda* and *Edwardsiella anguillarum* isolates. The MAR index values are shown using boxplots to summarize their central tendency and dispersion, with individual isolates overlaid as points to illustrate the distribution of resistance levels within and between species. The index is presented on a standardized scale ranging from 0 to 1.

The MAR index ranged from 0.00 to 0.44 in *E. tarda* (mean = 0.20) and from 0.00 to 0.68 in *E. anguillarum* (mean = 0.19). MAR index values ≥ 0.20 were observed in 11 of 19 *E. tarda* isolates (57.89%) and in 12 of 31 *E. anguillarum* isolates (38.71%). The highest MAR index value (0.68) was observed for *E. anguillarum* isolate BEP228. The distribution and individual variability of the MAR indices are shown in Figure 1.

Phenotypic screening for β-lactamase activity did not detect ESBL phenotypes in either species. One *E. anguillarum* isolate (BEP228) showed a phenotypic profile suggestive of ACBL production, which was reproducible in independent assays (Supplementary Figure S30).

## 4. Discussion

The present study was designed to establish pECVs for *E. anguillarum* isolated from Nile tilapia (*O. niloticus*) farmed in Brazil using the NRI approach. Considering the sample size obtained for *E. anguillarum*, the proposed pECVs provide a consistent framework for local epidemiological surveillance, allowing for discrimination between WT and NWT subpopulations. In contrast, the data generated for *E. tarda* should be interpreted as preliminary and exploratory, serving mainly to contextualize antibiotic resistance patterns within the genus rather than to support direct interspecies comparisons.

The importance of defining local pECVs is evident when considering the substantial heterogeneity in antimicrobial susceptibility profiles reported for *Edwardsiella* spp. across different geographic regions, host species, and production systems. Rahmawaty [4] demonstrated wide phenotypic and genotypic variability among *Edwardsiella* isolates from Taiwan, including both *E. tarda* and *E. anguillarum*, highlighting that resistance patterns are strongly impacted by local antimicrobial usage and ecological conditions. Similarly, Rocha [10] showed that pECVs derived from *E. tarda* varied according to fish species and farming systems in Brazil, reinforcing the need for species-specific epidemiological benchmarks rather than generalized cut-off values.

All isolates analyzed in the present study originated from Nile tilapia on commercial farms, a species typically produced under intensive farming conditions characterized by high stocking densities and frequent therapeutic interventions [1,7,10]. Such farming conditions favor antimicrobial exposure and sustained selective pressure, contributing to the emergence and maintenance of resistant phenotypes [7,10,13]. Thus, the pECVs proposed for *E. anguillarum* likely reflect the resistance distributions associated with intensive tilapia farming systems and should not be directly extrapolated to native fish species or low-input fish production systems [10,15,22]. This interpretation is supported by Reis et al. [15], who reported lower MAR indices and distinct resistance profiles in *E. tarda* isolated from tambaqui (*Colossoma macropomum*), a species generally reared under relatively less intensive conditions.

Although no unique intrinsic resistance patterns have been formally described for *Edwardsiella* spp., their profile is consistent with that of other *Enterobacterales*, characterized by natural resistance to narrow-spectrum penicillins and several non-β-lactam classes, while maintaining baseline susceptibility to most β-lactams [1]. In the members of this family, this basal resistance is primarily attributed to the structural features of β-lactamase genes, such as reduced outer membrane permeability, and their presence, with no basal chromosomal activity [9]. According to Nantongo [23], resistance to drugs such as oxacillin and penicillin in *E. tarda* is a typical family trait, often resulting from porin channels that restrict the entry of hydrophobic or large-molecular-weight molecules. Therefore, clinically significant β-lactam resistance observed in this genus likely reflects acquired mechanisms, such as the production of plasmid-mediated β-lactamases, e.g., blaTEM and blaCTX-M enzymes, coupled with alterations in membrane permeability rather than a uniquely elevated intrinsic resistance [9].

From a mechanistic perspective, the conserved susceptibility of the isolates to carbapenems, advanced-generation cephalosporins, aminoglycosides, and sulfamethoxazole/ trimethoprim is consistent with the modes of action of these antimicrobials [24]. Carbapenems and advanced cephalosporins inhibit bacterial cell-wall synthesis through high-affinity binding to penicillin-binding proteins and exhibit enhanced stability against most β-lactamases, whereas aminoglycosides target the 30S ribosomal subunit to inhibit protein synthesis [24]. Furthermore, the sulfamethoxazole/ trimethoprim combination acts as a folate pathway inhibitor, interfering with the synthesis of essential precursors for bacterial nucleic acid production [13,21]. Moreover, the observation that these isolates remained susceptible suggests that in this Brazilian aquaculture setting, broad-spectrum resistance mechanisms remain uncommon among *Edwardsiella* populations [10]. This interpretation is supported by recent studies in Brazil that reported a low frequency of -lactamase-associated resistance genes in *Edwardsiella* spp., in contrast to the high prevalence of ESBL-producing strains reported in other regions, such as Egypt and Asia [10].

In contrast, higher proportions of NWT isolates than those of WT isolates were observed for penicillins, fluoroquinolones, florfenicol, and tetracycline. Resistance to penicillins may be attributed to the basal chromosomal β-lactamase activity, reduced outer membrane permeability, or the presence of inhibitor-susceptible enzymes, as suggested by their variable responses to amoxicillin/ clavulanic acid [25]. The absence of ESBL phenotypes among all isolates, confirmed by phenotypic detection, is epidemiologically relevant and contrasts with reports from Egypt and parts of Asia, where the genotypic detection of *bla_CTX-M* and *bla_TEM* in *E. tarda* exceeded 80% of the isolates [1]. These findings suggest that, although resistance to penicillins and some cephalosporins is present, ESBLs are not yet widespread among Brazilian *Edwardsiella* isolates [10].

Therefore, resistance to cephalosporins such as ceftriaxone and aztreonam, even in the absence of ESBL production, may be associated with alternative mechanisms. Similar patterns have been described in *Edwardsiella* spp., including *E. tarda*, isolated from ornamental fish and humans. In such species, mutations affecting porin proteins (OmpF/OmpC) and overexpression of efflux pumps (AcrAB–TolC) contributed to intermediate resistance levels without detectable ESBL genes [11,25,26]. These resistance mechanisms are compatible with the heterogeneous inhibition zone distributions observed in the present study.

Only one *E. anguillarum* isolate exhibited a phenotypic profile suggestive of ACBL production. Although this result was reproducible across independent assays, its exclusive phenotypic nature requires cautious interpretation and molecular confirmation to determine the genetic basis in this case. Rocha et al. [10] reported a low frequency of β-lactamase-associated resistance genes in *Edwardsiella* spp., supporting the view that enzymatic β-lactam resistance remains limited in Brazilian aquaculture; however, its emergence cannot be ruled out under sustained selective pressure.

Fluoroquinolone resistance displayed marked heterogeneity among *E. anguillarum* isolates, reflecting the diversity of the mechanisms involved in resistance to this antimicrobial class. Fluoroquinolones inhibit DNA gyrase and topoisomerase IV, and resistance may arise through point mutations in quinolone resistance-determining regions, plasmid-mediated quinolone resistance genes (*qnrA* and *qnrS*), efflux pump overexpression, or reduced outer-membrane permeability [1,13]. The coexistence of WT and NWT subpopulations observed in the present study suggests continuous selection and dissemination of resistance determinants within tilapia farming systems, as indicated by other studies [3,10].

Resistance to florfenicol and tetracycline deserves particular attention as these are the only antimicrobials officially approved for therapeutic use in fish in Brazil [27]. Both compounds inhibited bacterial protein synthesis by targeting the 50S and 30S ribosomal subunits. Resistance to these classes is commonly mediated by transferable genes such as *floR*, *fexA*, and *tet* variants, which are frequently located on plasmids, transposons, and other mobile genetic elements [1,10]. Therefore, the intermediate WT/NWT distributions observed for *E. anguillarum* are consistent with the active role of horizontal gene transfer in shaping resistance profiles in aquaculture environments [3].

The MAR index and high frequency of MDR isolates observed in *E. anguillarum* further indicate sustained selective pressures in intensive tilapia farming systems. Similar MAR thresholds associated with high-risk environments have been reported in other studies involving *Edwardsiella* spp. [5,10]. Notably, the presence of MDR isolates with relatively low MAR values highlights heterogeneous resistance profiles arising from different combinations of resistant antimicrobial classes rather than from the uniform accumulation of resistance mechanisms.

From a One Health perspective, the implications of these findings extend beyond fish health. Aquaculture environments provide favorable conditions, including high bacterial densities, biofilm formation, and exposure to antimicrobial residues at sub-inhibitory concentrations, for horizontal gene transfer [3]. *Edwardsiella* is recognized as a core member of the aquatic resistome owing to its abundance and ability to harbor diverse antimicrobial resistance genes (ARGs) on chromosomes and plasmids [3]. Furthermore, it serves as a crucial indicator organism for monitoring the accumulation of ARGs in aquatic habitats, acting as a reservoir for detecting resistance determinants in the environment and transmitting them to the relatively broader microbiome [3]. These genes may be exchanged for opportunistic human pathogens, contributing to the expansion of environmental resistomes and posing substantial risks to animal, environmental, and human health.

These results highlight the dynamic interplay between antimicrobial usage, resistance mechanisms, and environmental factors in aquaculture while emphasizing the importance of integrated phenotypic and molecular surveillance strategies aligned with the One Health principle.

## 5. Conclusions

In this study, pECVs for *E. anguillarum* isolated from Nile tilapia (*O. niloticus*) in Brazil were determined, providing a practical phenotypic framework for distinguishing between WT and NWT populations using NRI. In addition, the data presented herein provide preliminary information on the antibacterial resistance of *E. tarda* strains and other relevant fish pathogens. Brazilian *Edwardsiella* lineages appear to be at an intermediate stage of antimicrobial resistance dissemination, with their heterogeneous susceptibility profiles shaped by selective pressure in intensive tilapia farming. Although most isolates remain susceptible to several antimicrobial classes, the presence of NWT and MDR isolates, with MAR indices ≥0.2, observed across regions and years, indicates ongoing selection driven by antimicrobial use. Finally, this study makes available, and subject to comparison, relevant data on antibacterial resistance and local pECVs, and provides preliminary insights into the two major fish pathogens of the genus *Edwardsiella*.

## Figures and Tables

**Table 1:** Identification of *Edwardsiella* spp. isolates used in this study, indicating the host, state of origin in Brazil, and year of isolation.

Isolate	Host	State	Year
BEP76	*Oreochromis niloticus*	Paraná	2017
BEP77	*Oreochromis niloticus*	Paraná	2017
BEP89	*Oreochromis niloticus*	Paraná	2017
BEP93	*Oreochromis niloticus*	Paraná	2017
BEP105	*Oreochromis niloticus*	Paraná	2017
BEP170	*Oreochromis niloticus*	São Paulo	2019
BEP179	*Oreochromis niloticus*	Paraná	2020
BEP203	*Oreochromis niloticus*	Paraná	2020
BEP214	*Oreochromis niloticus*	Paraná	2020
BEP219	*Oreochromis niloticus*	Paraná	2020
BEP228	*Oreochromis niloticus*	Minas Gerais	2020
BEP237	*Oreochromis niloticus*	Paraná	2020
BEP239	*Oreochromis niloticus*	Not informed	2020
BEP257	*Oreochromis niloticus*	Not informed	2020
BEP258	*Oreochromis niloticus*	Not informed	2020
BEP266	*Oreochromis niloticus*	Piauí	2020
BEP273	*Oreochromis niloticus*	Not informed	2020
BEP282	*Oreochromis niloticus*	Paraná	2021
BEP283	*Oreochromis niloticus*	Paraná	2021
BEP286	*Oreochromis niloticus*	Not informed	2021
BEP287	*Oreochromis niloticus*	São Paulo	2021
BEP288	*Oreochromis niloticus*	Paraná	2021
BEP289	*Oreochromis niloticus*	Paraná	2021
BEP297	*Oreochromis niloticus*	Paraná	2021
BEP300	*Oreochromis niloticus*	São Paulo	2021
BEP304	*Oreochromis niloticus*	São Paulo	2021
BEP318	*Oreochromis niloticus*	Bahia	2021
BEP319	*Oreochromis niloticus*	Paraná	2021
BEP320	*Oreochromis niloticus*	Not informed	2021
BEP334	*Oreochromis niloticus*	Paraná	2021
BEP335	*Oreochromis niloticus*	Not informed	2021
BEP336	*Oreochromis niloticus*	Not informed	2021
BEP359	*Oreochromis niloticus*	Paraná	2022
BEP426	*Oreochromis niloticus*	Paraná	2023
BEP435	*Oreochromis niloticus*	Paraná	2024
BEP436	*Oreochromis niloticus*	Not informed	2024
BEP450	*Oreochromis niloticus*	Paraná	2024
BEP466	*Oreochromis niloticus*	Maranhão	2024
BEP512	*Oreochromis niloticus*	Piauí	2025
BEP513	*Oreochromis niloticus*	Piauí	2025
BEP523	*Oreochromis niloticus*	São Paulo	2025
BEP525	*Oreochromis niloticus*	Ceará	2025
BEP554	*Oreochromis niloticus*	Piauí	2025
BEP561	*Oreochromis niloticus*	Paraná	2025
SAD1	*Oreochromis niloticus*	São Paulo	2023
SAD2	*Oreochromis niloticus*	São Paulo	2023
SAD3	*Oreochromis niloticus*	São Paulo	2023
2259.2	*Oreochromis niloticus*	São Paulo	2023
5631.2	*Oreochromis niloticus*	Minas Gerais	2024

**Table 2:** Phenotypic antimicrobial susceptibility profiles of *Edwardsiella tarda* and *Edwardsiella anguillarum* from Nile tilapia (*Oreochromis niloticus*) to β-lactam.

Antimicrobial	*Edwardsiella tarda*							*Edwardsiella anguillarum*						
	Min	Max	Mean	SD	pECV	WT (%)	NWT (%)	Min	Max	Mean	SD	pECV	WT (%)	NWT (%)
β-lactams														
*Penicillins*														
Amoxicillin	6	30	26.7	2	20	58%	42%	6	33	27.1	2.44	20	97%	3%
Ampicillin	6	32	28.8	3	22	58%	42%	6	38	30.5	3.41	21	97%	3%
Amoxicillin + Clavulanic acid	22	45	44	2	38	32%	68%	12	50	44.0	5.17	31	42%	58%
Piperacillin + Tazobactam	25	36	32.5	2	26	95%	5%	12	43	38.8	3.35	30	81%	19%
*Cephalosporins*														
Cefazolin	6	32	25.5	3	18	95%	5%	6	43	26.8	5.64	12	97%	3%
Cephalexin	16	27	23.8	2	19	95%	5%	6	32	29.7	2.97	22	52%	48%
Cefoxitin	14	34	31.8	2	27	89%	11%	6	40	37.7	4.64	26	90%	10%
Cefotaxime	22	41	38.1	4	28	89%	11%	30	46	41.7	2.82	34	81%	19%
Ceftriaxone	20	42	36.8	3	28	89%	11%	26	46	39.3	2.82	32	90%	10%
Ceftazidime	18	37	30.7	3	22	95%	5%	20	45	38.4	3.80	28	94%	6%
Cefepime	24	36	32.8	4	23	100%	0%	17	42	36.0	3.34	27	94%	6%
Ceftiofur	22	40	33	4	22	100%	0%	23	40	35.7	2.93	28	97%	3%
Cefuroxime	17	35	31.3	3	22	89%	11%	23	40	39.0	3.95	29	81%	19%
*Carbapenems*														
Imipenem	24	38	33.2	3	25	89%	11%	27	42	35.4	3.84	25	100%	0%
Meropenem	21	38	32.4	4	22	95%	5%	21	45	38.3	3.92	28	90%	10%
*Monobactams*														
Aztreonam	16	46	32.8	6	16	100%	0%	6	50	44.8	3.58	35	81%	19%

Min, minimum inhibition zone diameter (mm); Max, maximum inhibition zone diameter (mm); Mean, mean inhibition zone diameter (mm); SD, standard deviation; pECV, provisional local epidemiological cut-off value; WT, percentage of wild-type strains; NWT, percentage of non-wild-type strains.

**Table 3:** Phenotypic antimicrobial susceptibility profiles of *Edwardsiella tarda* and *Edwardsiella anguillarum* isolates from Nile tilapia (*Oreochromis niloticus*) to aminoglycosides, fluoroquinolones, tetracyclines, phenicols, and folate pathway inhibitors.

Antimicrobial	*Edwardsiella tarda*							*Edwardsiella anguillarum*						
	Min	Max	Mean	SD	pECV	WT (%)	NWT (%)	Min	Max	Mean	SD	pECV	WT (%)	NWT (%)
Aminoglycosides														
Amikacin	6	28	18.9	3.5	10	95%	5%	17	31	23.3	4.60	11	100%	0%
Gentamicin	6	24	22	2	16	84%	16%	19	29	23.1	2.69	16	100%	0%
Tobramycin	16	25	21.7	3.5	12	100%	0.0%	13	29	24.2	2.13	18	97%	3%
Streptomycin	10	26	24.2	2.6	17	74%	26	9	38	25.9	4.20	15	94%	6%
Fluoroquinolones														
Ciprofloxacin	12	40	30.2	5.9	15	95%	5%	26	54	51.0	2.17	45	58%	42%
Enrofloxacin	22	39	36.6	3.8	26	37%	46.7%	24	44	39.9	1.30	36	71%	29%
Levofloxacin	26	42	39.6	2.9	32	42%	58%	27	50	47.2	5.88	32	90%	10%
Norfloxacin	18	41	40	1.4	36	11%	89%	24	50	48.1	5.73	33	77%	23%
Marbofloxacin	24	33	27.9	3	20	100%	0.0%	24	50	44.0	3.59	34	84%	16%
Tetracyclines														
Tetracycline	6	30	-	-	-	-	-	6	36	32.0	2.60	25	52%	48%
Phenicols														
Florfenicol	10	40	35.4	2.7	28	53%	47%	11	42	36.7	4.70	24	52%	48%
Folate pathway inhibitors														
Sulfamethoxazole + trimethoprim	6	44	35.7	5	23	95%	5%	23	52	35.3	5.48	21	100%	0%

Min, minimum inhibition zone diameter (mm); Max, maximum inhibition zone diameter (mm); Mean, mean inhibition zone diameter (mm); SD, standard deviation; pECV, provisional local epidemiological cut-off value; WT, percentage of wild-type strains; NWT, percentage of non-wild-type strains.

## Data Availability

The original contributions presented in this study are included in the article/Supplementary Material. Further inquiries can be directed to the corresponding author.

## Supplementary Materials

The following supporting information can be downloaded at: https://www.mdpi.com/article/10.3390/microorganisms14030523/s1, Figure S1: NRI analysis of amikacin inhibition zone diameters for *Edwardsiella anguillarum* and *Edwardsiella tarda*; Figure S2: NRI analysis of amoxicillin + clavulanic acid inhibition zone diameters for *Edwardsiella anguillarum* and *Edwardsiella tarda*; Figure S3: NRI analysis of amoxicillin inhibition zone diameters for *Edwardsiella anguillarum* and *Edwardsiella tarda*; Figure S4: NRI analysis of ampicillin inhibition zone diameters for *Edwardsiella anguillarum* and *Edwardsiella tarda*; Figure S5: NRI analysis of aztreonam inhibition zone diameters for *Edwardsiella anguillarum* and *Edwardsiella tarda*; Figure S6: NRI analysis of cefazolin inhibition zone diameters for *Edwardsiella anguillarum* and *Edwardsiella tarda*; Figure S7: NRI analysis of cefepime inhibition zone diameters for *Edwardsiella anguillarum* and *Edwardsiella tarda*; Figure S8: NRI analysis of cefotaxime inhibition zone diameters for *Edwardsiella anguillarum* and *Edwardsiella tarda*; Figure S9: NRI analysis of cefoxitin inhibition zone diameters for *Edwardsiella anguillarum* and *Edwardsiella tarda*; Figure S10: NRI analysis of ceftazidime inhibition zone diameters for *Edwardsiella anguillarum* and *Edwardsiella tarda*; Figure S11: NRI analysis of ceftiofur inhibition zone diameters for *Edwardsiella anguillarum* and *Edwardsiella tarda*; Figure S12: NRI analysis of ceftriaxone inhibition zone diameters for *Edwardsiella anguillarum* and *Edwardsiella tarda*; Figure S13: NRI analysis of cefuroxime inhibition zone diameters for *Edwardsiella anguillarum* and *Edwardsiella tarda*; Figure S14: NRI analysis of cephalexin inhibition zone diameters for *Edwardsiella anguillarum* and *Edwardsiella tarda*; Figure S15: NRI analysis of ciprofloxacin inhibition zone diameters for *Edwardsiella anguillarum* and *Edwardsiella tarda*; Figure S16: NRI analysis of enrofloxacin inhibition zone diameters for *Edwardsiella anguillarum* and *Edwardsiella tarda*; Figure S17: NRI analysis of florfenicol inhibition zone diameters for *Edwardsiella anguillarum* and *Edwardsiella tarda*; Figure S18: NRI analysis of gentamicin inhibition zone diameters for *Edwardsiella anguillarum* and *Edwardsiella tarda*; Figure S19: NRI analysis of imipenem inhibition zone diameters for *Edwardsiella anguillarum* and *Edwardsiella tarda*; Figure S20: NRI analysis of levofloxacin inhibition zone diameters for *Edwardsiella anguillarum* and *Edwardsiella tarda*; Figure S21: NRI analysis of marbofloxacin inhibition zone diameters for *Edwardsiella anguillarum* and *Edwardsiella tarda*; Figure S22: NRI analysis of meropenem inhibition zone diameters for *Edwardsiella anguillarum* and *Edwardsiella tarda*; Figure S23: NRI analysis of norfloxacin inhibition zone diameters for *Edwardsiella anguillarum* and *Edwardsiella tarda*; Figure S24: NRI analysis of piperacillin + tazobactam inhibition zone diameters for *Edwardsiella anguillarum* and *Edwardsiella tarda*; Figure S25: NRI analysis of streptomycin inhibition zone diameters for *Edwardsiella anguillarum* and *Edwardsiella tarda*; Figure S26: NRI analysis of sulfamethoxazole/trimethoprim inhibition zone diameters for *Edwardsiella anguillarum* and *Edwardsiella tarda*; Figure S27: NRI analysis of tetracycline inhibition zone diameters for *Edwardsiella anguillarum* and *Edwardsiella tarda*; Figure S28: NRI analysis of tobramycin inhibition zone diameters for *Edwardsiella anguillarum* and *Edwardsiella tarda*; Table S1: Inhibition zone diameters (mm) obtained for the quality control strain *Escherichia coli* ATCC 25922 used to validate antimicrobial susceptibility testing; Figure S29: Internal quality control plates confirming the performance of phenotypic assays for AmpC β-lactamase and ESBL detection; Figure S30: Phenotypic detection of AmpC β-lactamase in the *Edwardsiella anguillarum* BEP228 isolate, showing disk diffusion profiles suggestive of AmpC β-lactamase production.

## Abbreviations

The following abbreviations are used in this manuscript:

AmpC	Ampicillin cephalosporinase
ARGs	Antimicrobial resistance genes
ATCC	American Type Culture Collection
BEP	Bacteria isolated from fish
BrCAST	Brazilian Committee on Antimicrobial Susceptibility Testing
CLSI	Clinical and Laboratory Standards Institute
DNA	Deoxyribonucleic acid
ECV	Epidemiological cut-off value
ESBL	Extended-spectrum β-lactamase
EUCAST	European Committee on Antimicrobial Susceptibility Testing
MAR	Multiple antibiotic resistance
MDR	Multidrug-resistant
MHBA	Mueller–Hinton blood agar
NRI	Normalized resistance interpretation
NWT	Non-wild type
pECV	Provisional local epidemiological cut-off value
WT	Wild type
