# Supplementary material for: Phenotypic Antimicrobial Resistance Profiles and Provisional Epidemiological Cut-Off Values of Edwardsiella anguillarum Isolated from Farmed Nile Tilapia (Oreochromis niloticus) in Brazil, with Exploratory Data on Edwardsiella tarda
Source: Microorganisms. 2026 Feb 24;14(3):523. doi: 10.3390/microorganisms14030523 (PMC13029526; doi:10.3390/microorganisms14030523)
Supplement: Supplementary file 1 [file microorganisms-14-00523-s001.zip › microorganisms-3978187-supplementary.pdf]

Article

Phenotypic Antimicrobial Resistance Profiles and Provisional Epidemiological Cut-off Values of *Edwardsiella anguillarum* Isolated from Farmed Nile Tilapia (*Oreochromis niloticus*) in Brazil, with Exploratory Data on *Edwardsiella tarda*

Natália Amoroso Ferrari [1], Vittória Cueva Segura da Silva [1], Pamela Giovana Turini [1], Julia Faria de Souza [1], Raffaella Menegueti Mainardi [1], Mayza Brandão da Silva [1], Alene Santos Souza [1], Gabriel Diogo Guimarães [1], Maisa Fabiana Menck-Costa [1], Marco Rozas-Serri [2], Mariene Miyoko Natori [2], Renata Galetti [2] and Ulisses de Padua Pereira [1,*]

1 Laboratory of Fish Bacteriology, Department of Preventive Veterinary Medicine, State University of
Londrina, Londrina 86057-970, Brazil; natalia.amoroso@uel.br (N.A.F.); vittoria.cuevas@uel.br (V.C.S.d.S.); pamelaturini@gmail.com (P.G.T.); juliafaria.souza@uel.br (J.F.d.S.);
raffaellammveter@gmail.com (R.M.M.); brandao.mayza@gmail.com (M.B.d.S.); alenesantos47@gmail.com (A.S.S.); gdiogo705@gmail.com (G.D.G.); maisa.menckcosta@uel.br (M.F.M.-C.)

2 Pathovet Laboratory, Ribeirão Preto 14025-020, Brazil; marco.rozas@pathovet.cl (M.R.-S.); mariene.natori@pathovet.cl (M.M.N.); renata.galetti@pathovet.cl (R.G.)

* Correspondence: upaduapereira@uel.br; Tel.: +55-43-33714259

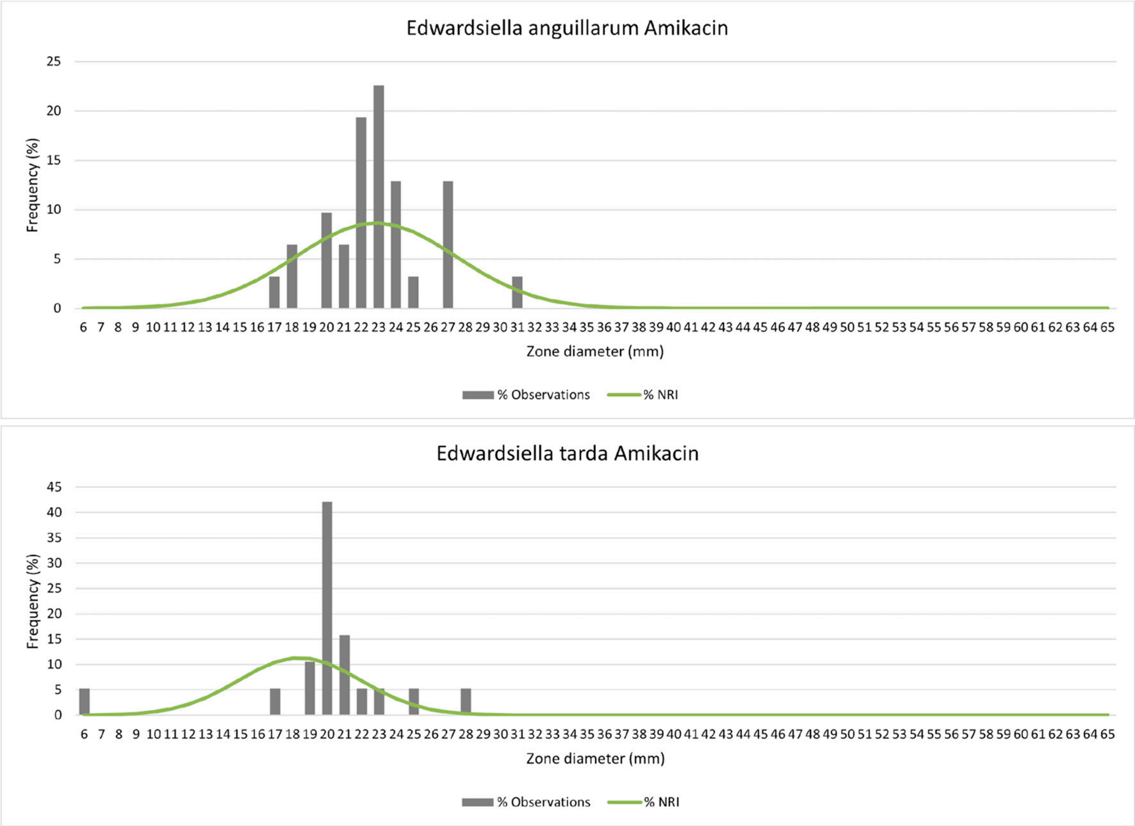

Supplementary Figure S1. NRI analysis of amikacin inhibition zone diameters for *Edwardsiella anguillarum* and *Edwardsiella tarda*

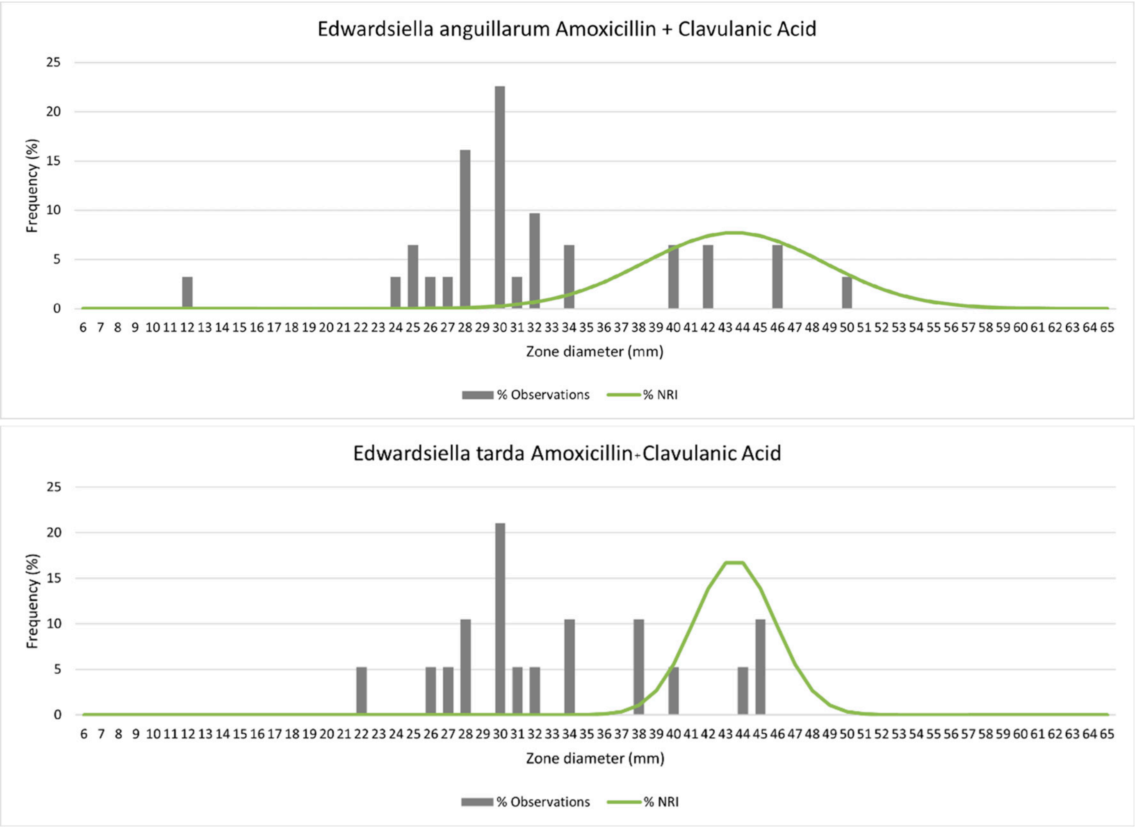

Supplementary Figure S2. NRI analysis of amoxicillin + clavulanic acid inhibition zone diameters for *Edwardsiella anguillarum* and *Edwardsiella tarda*

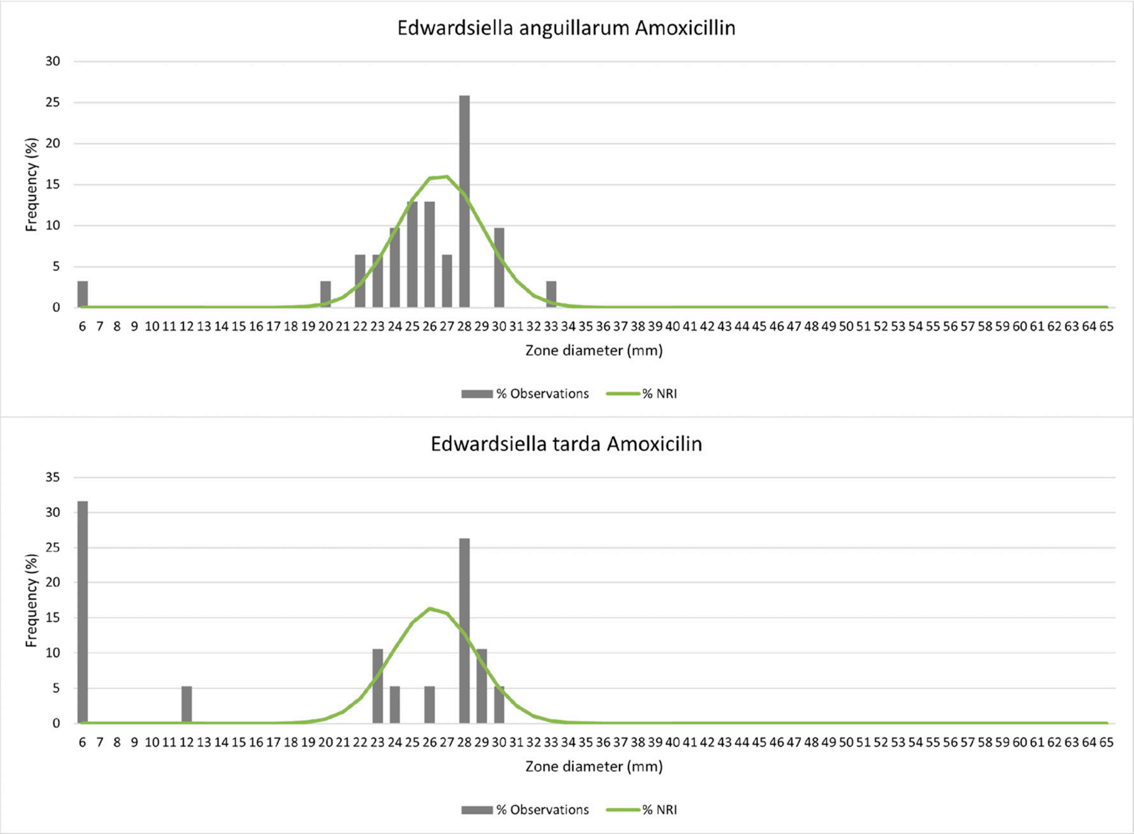

Supplementary Figure S3. NRI analysis of amoxicillin inhibition zone diameters for *Edwardsiella anguillarum* and *Edwardsiella tarda*

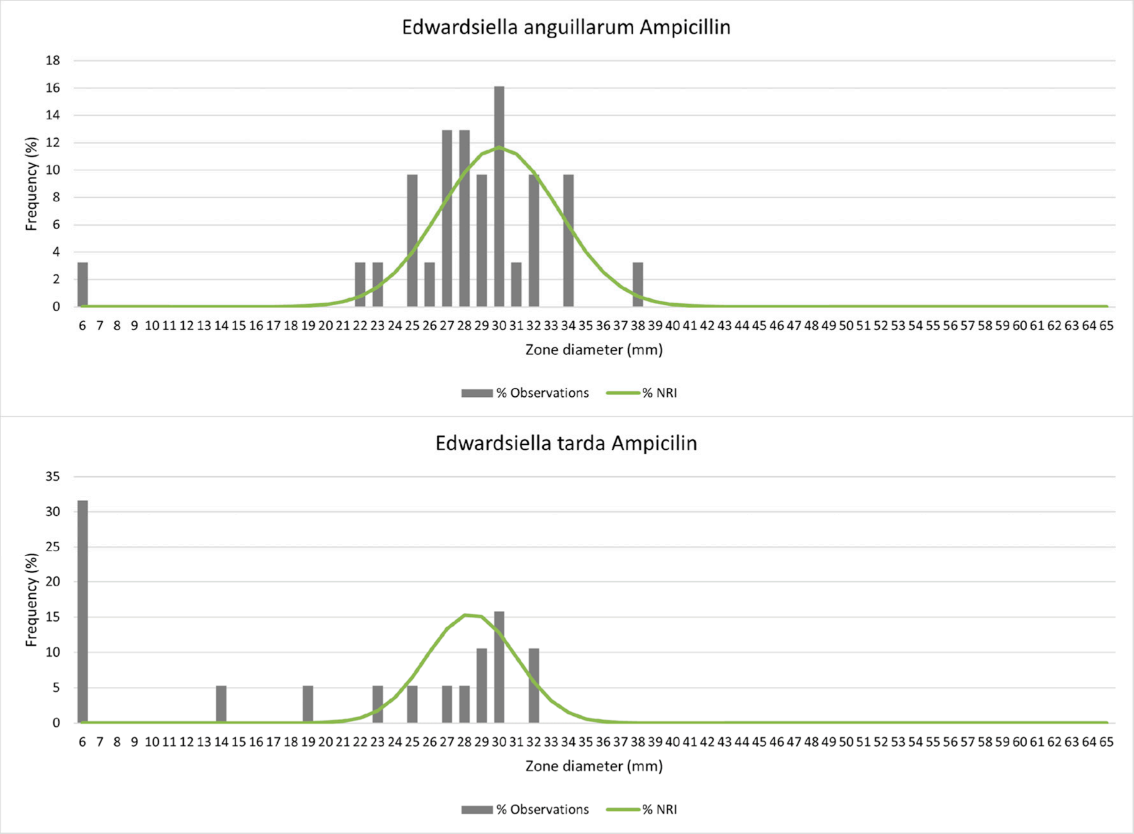

Supplementary Figure S4. NRI analysis of ampicillin inhibition zone diameters for *Edwardsiella anguillarum* and *Edwardsiella tarda*

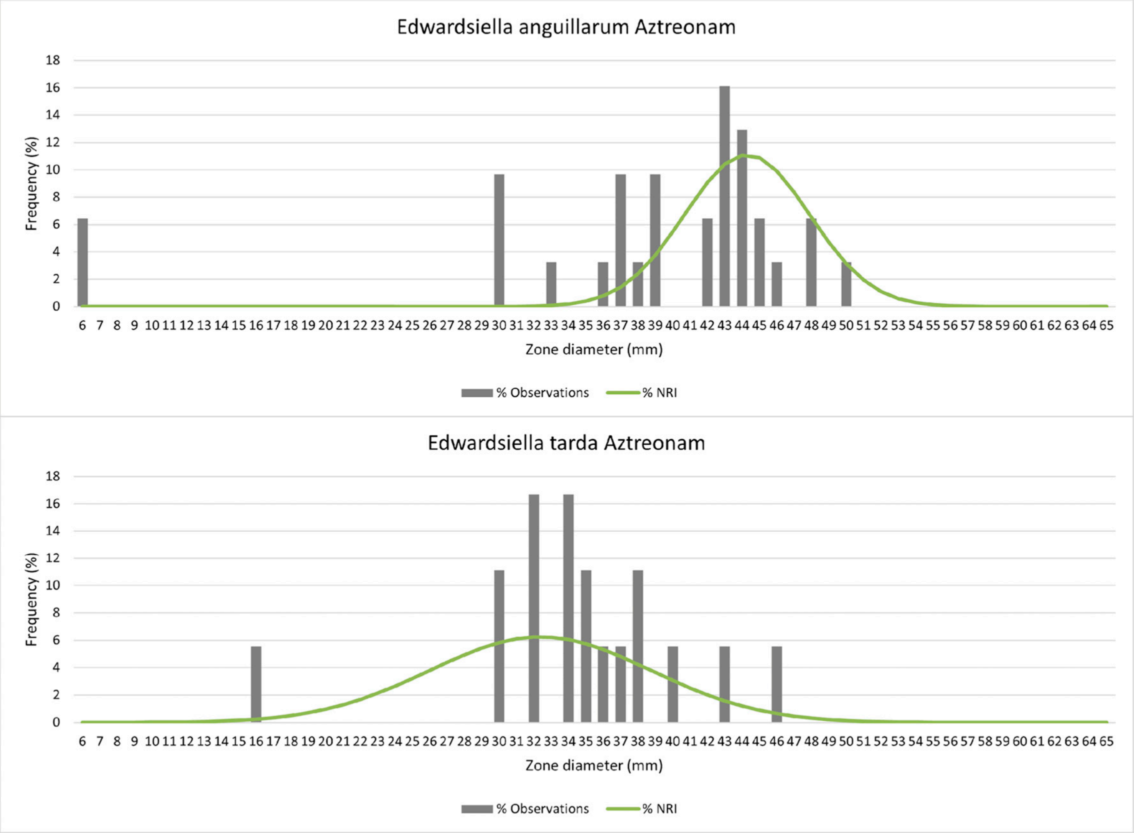

Supplementary Figure S5. NRI analysis of aztreonam inhibition zone diameters for *Edwardsiella anguillarum* and *Edwardsiella tarda*

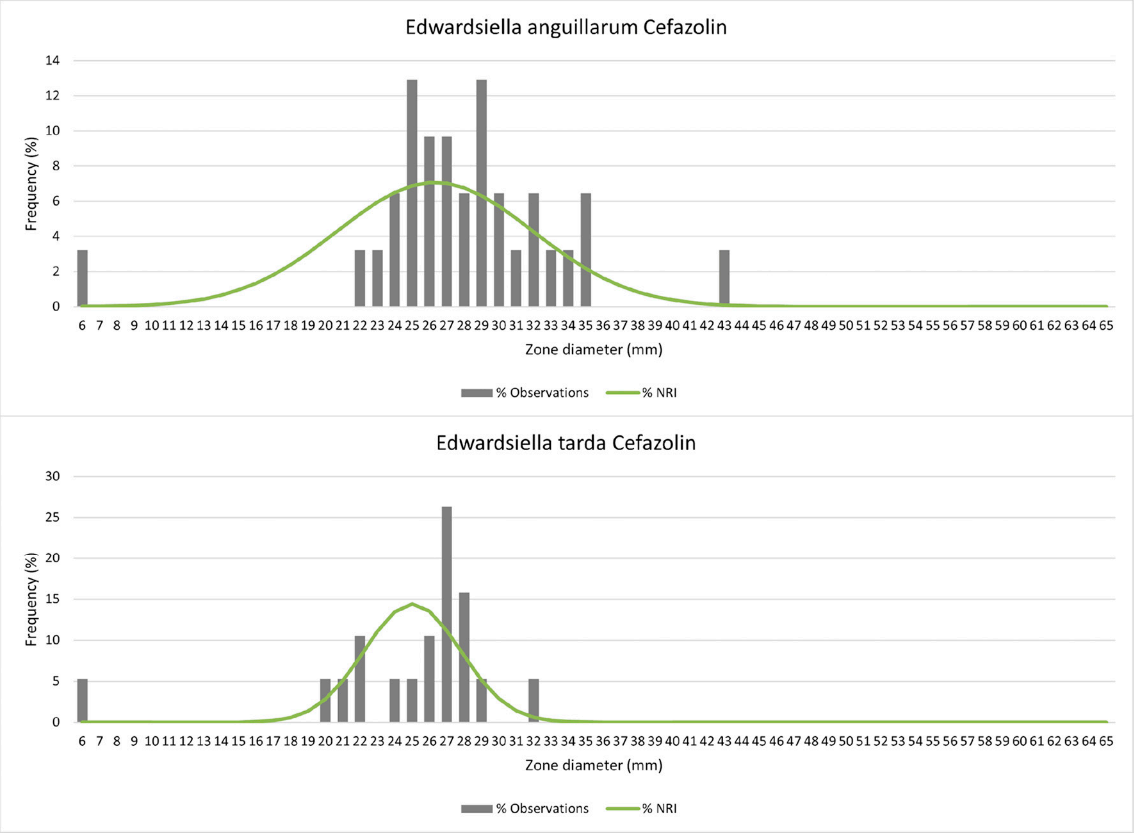

Supplementary Figure S6. NRI analysis of cefazolin inhibition zone diameters for *Edwardsiella anguillarum* and *Edwardsiella tarda*

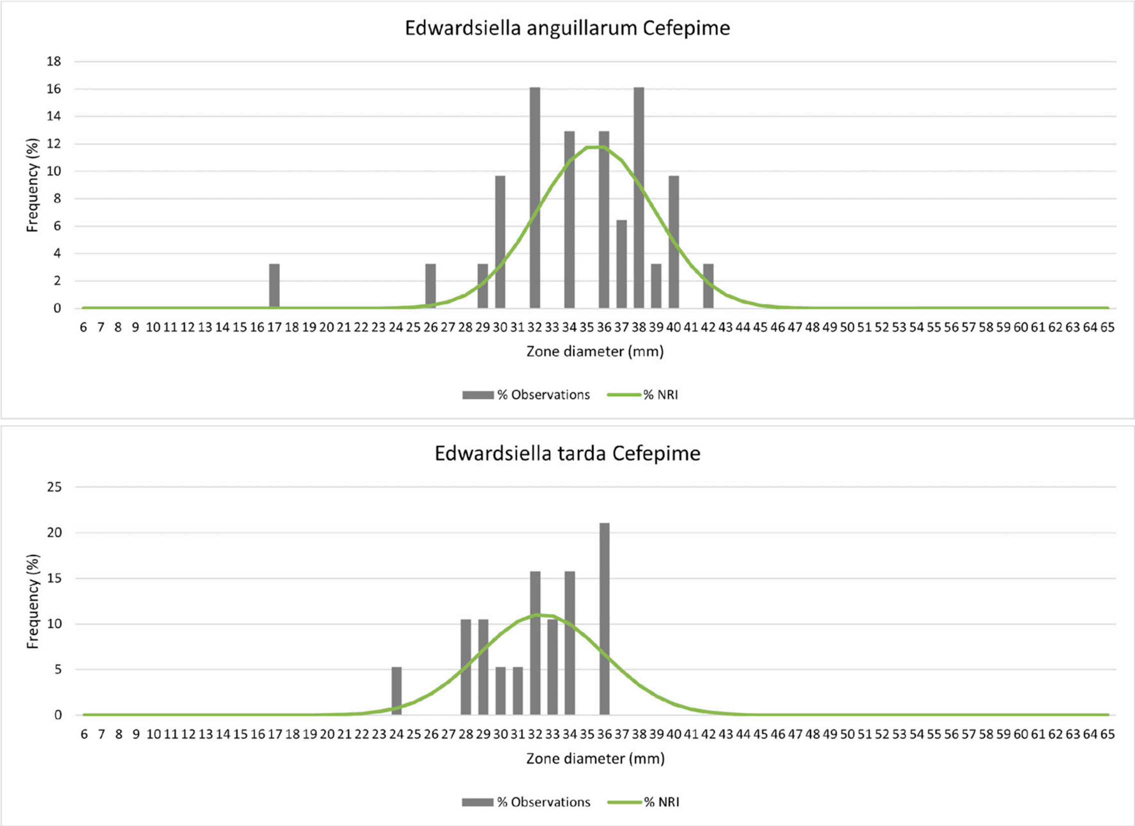

Supplementary Figure S7. NRI analysis of cefepime inhibition zone diameters for *Edwardsiella anguillarum* and *Edwardsiella tarda*

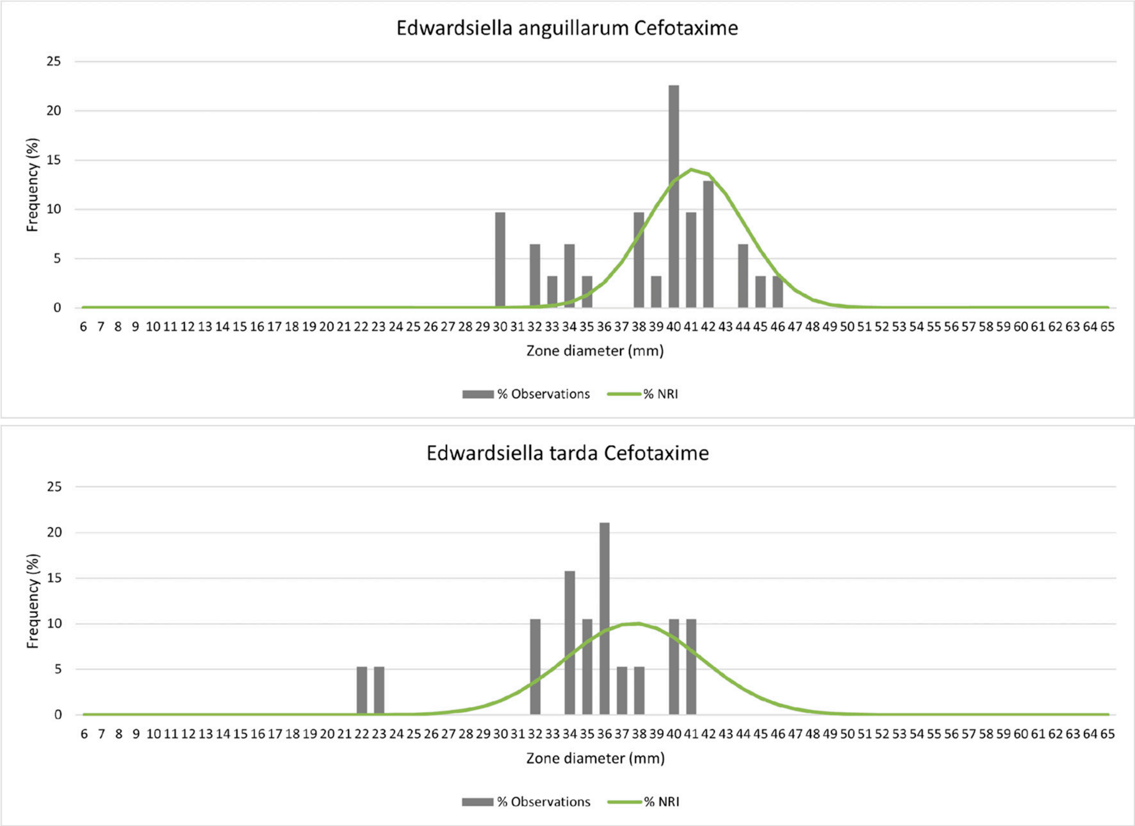

Supplementary Figure S8. NRI analysis of cefotaxime inhibition zone diameters for *Edwardsiella anguillarum* and *Edwardsiella tarda*

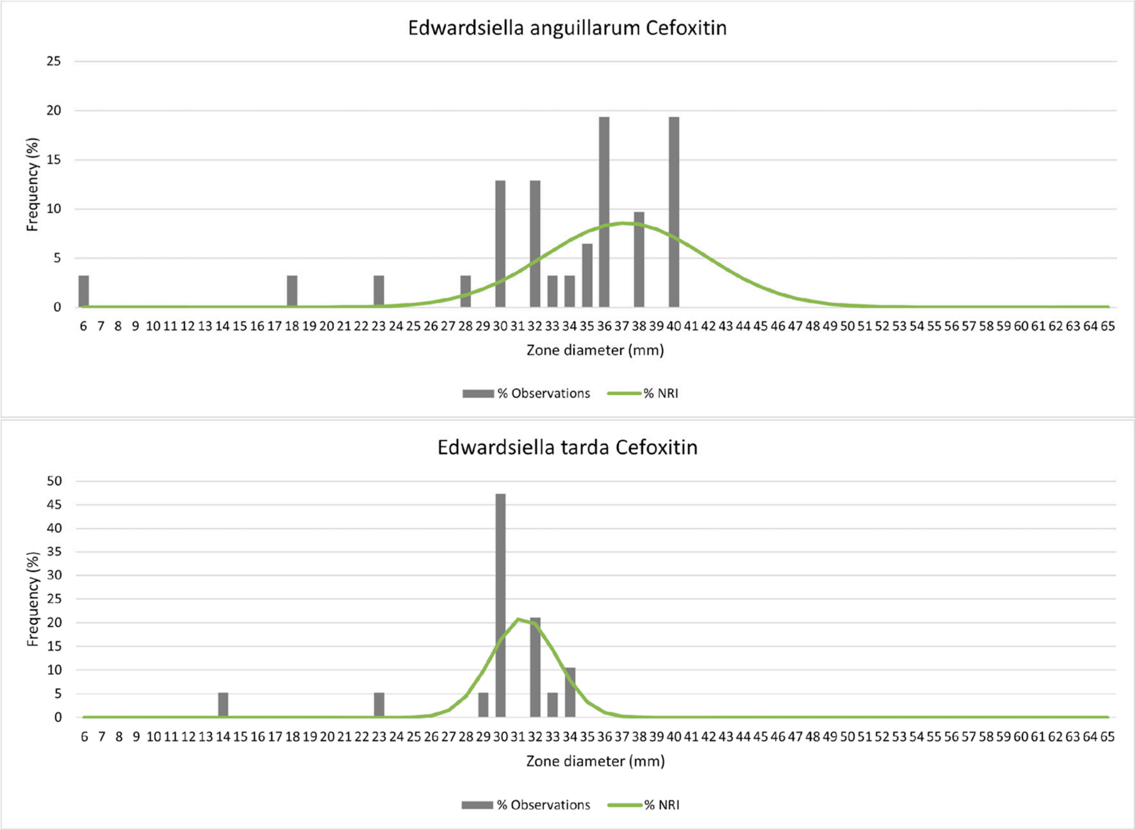

Supplementary Figure S9. NRI analysis of cefoxitin inhibition zone diameters for *Edwardsiella anguillarum* and *Edwardsiella tarda*

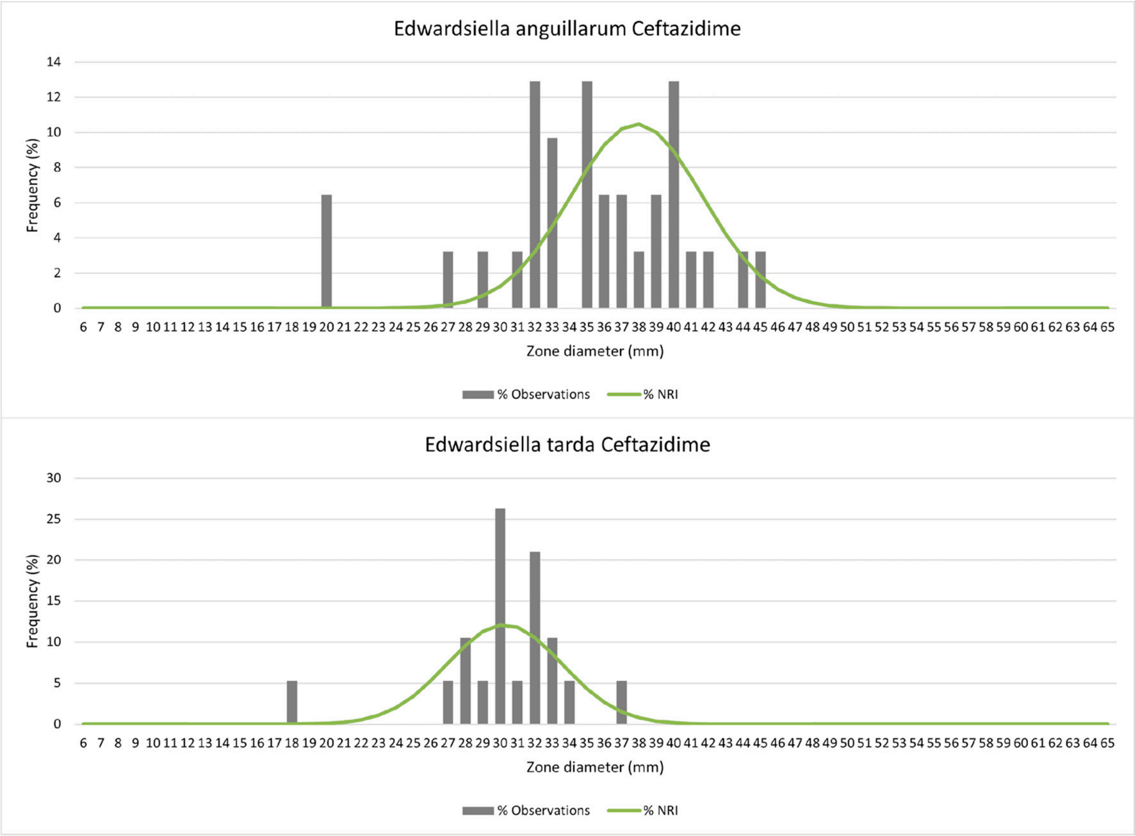

Supplementary Figure S10. NRI analysis of ceftazidime inhibition zone diameters for *Edwardsiella anguillarum* and *Edwardsiella tarda*

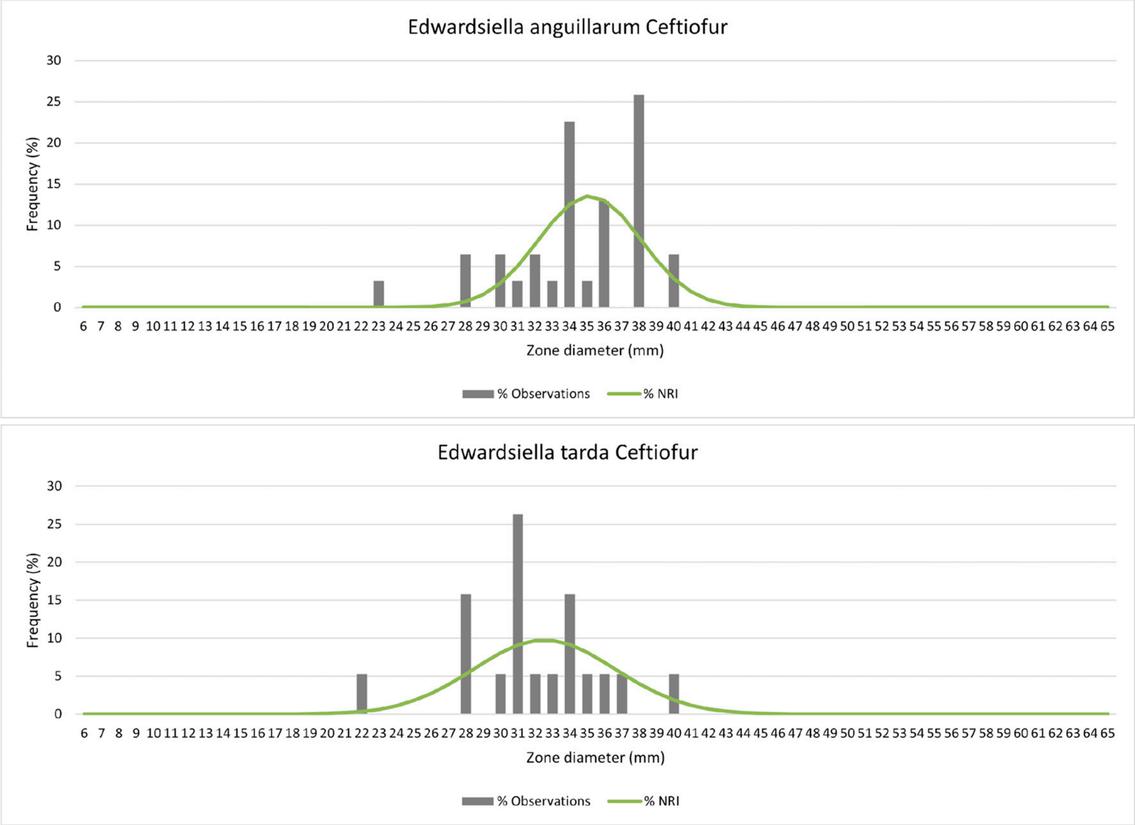

Supplementary Figure S11. NRI analysis of ceftiofur inhibition zone diameters for *Edwardsiella anguillarum* and *Edwardsiella tarda*

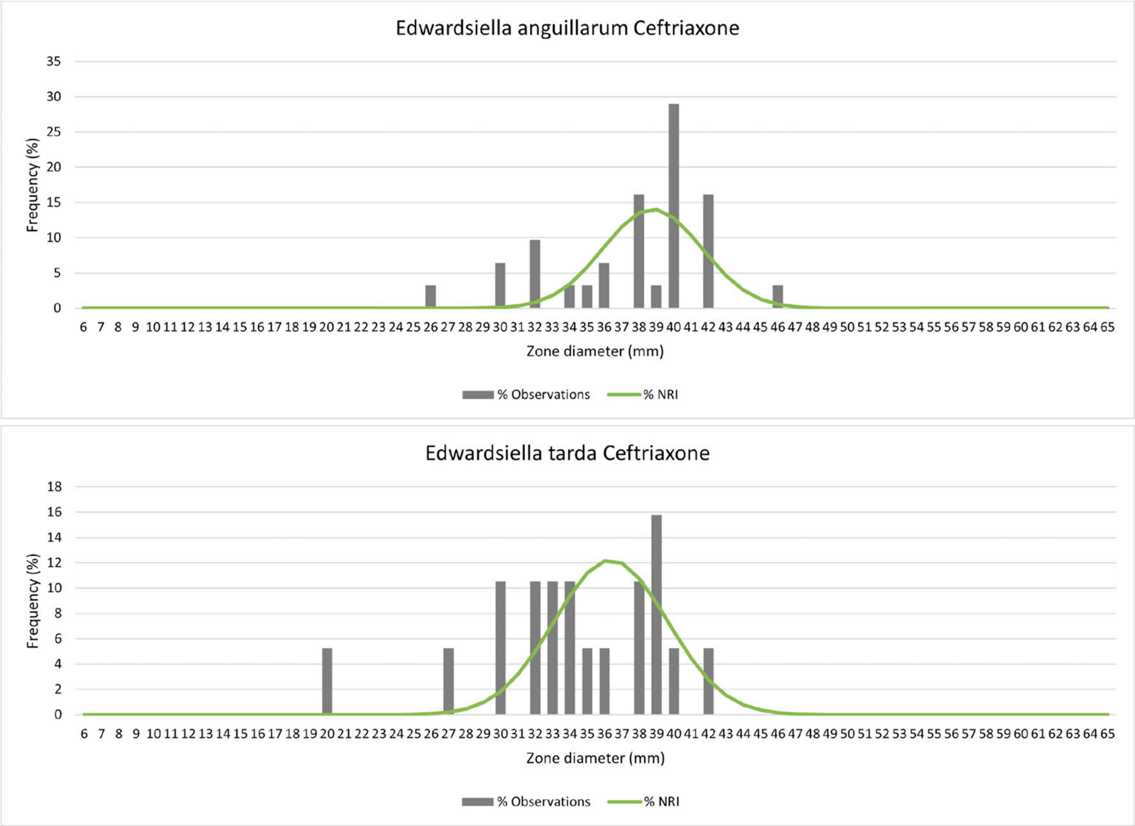

Supplementary Figure S12. NRI analysis of ceftriaxone inhibition zone diameters for *Edwardsiella anguillarum* and *Edwardsiella tarda*

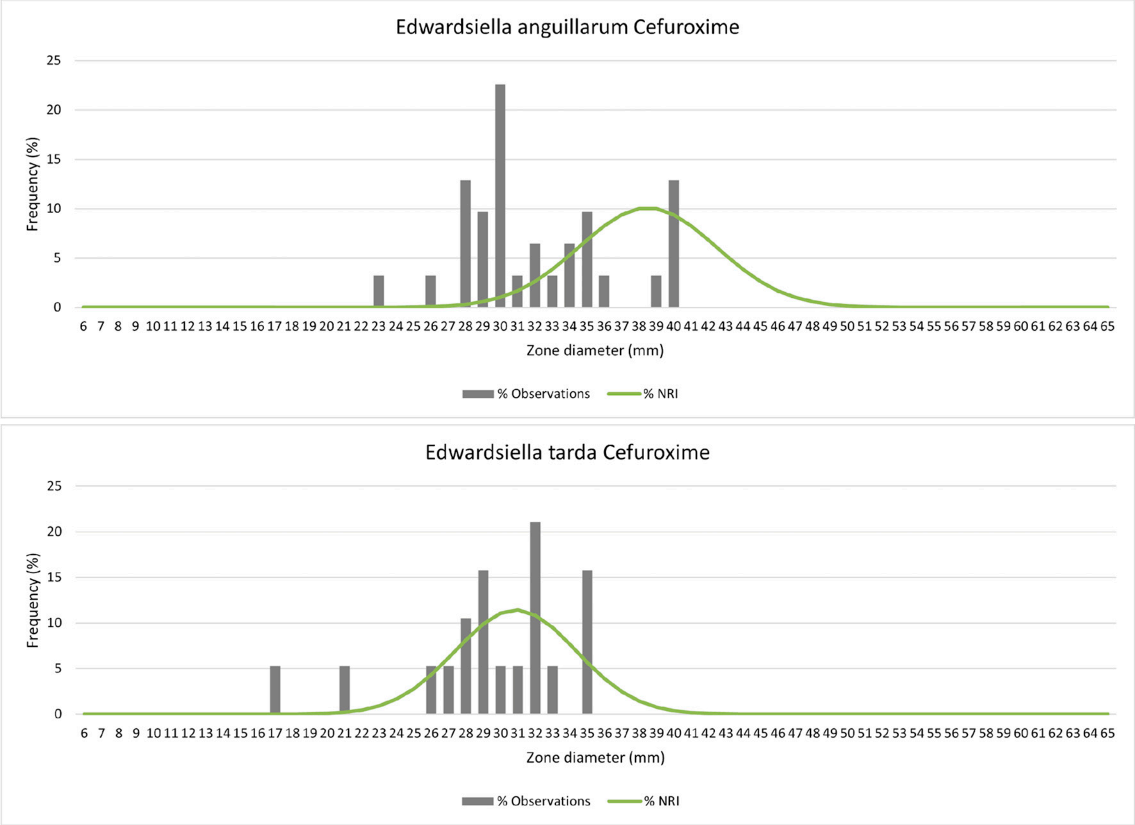

Supplementary Figure S13. NRI analysis of cefuroxime inhibition zone diameters for *Edwardsiella anguillarum* and *Edwardsiella tarda*

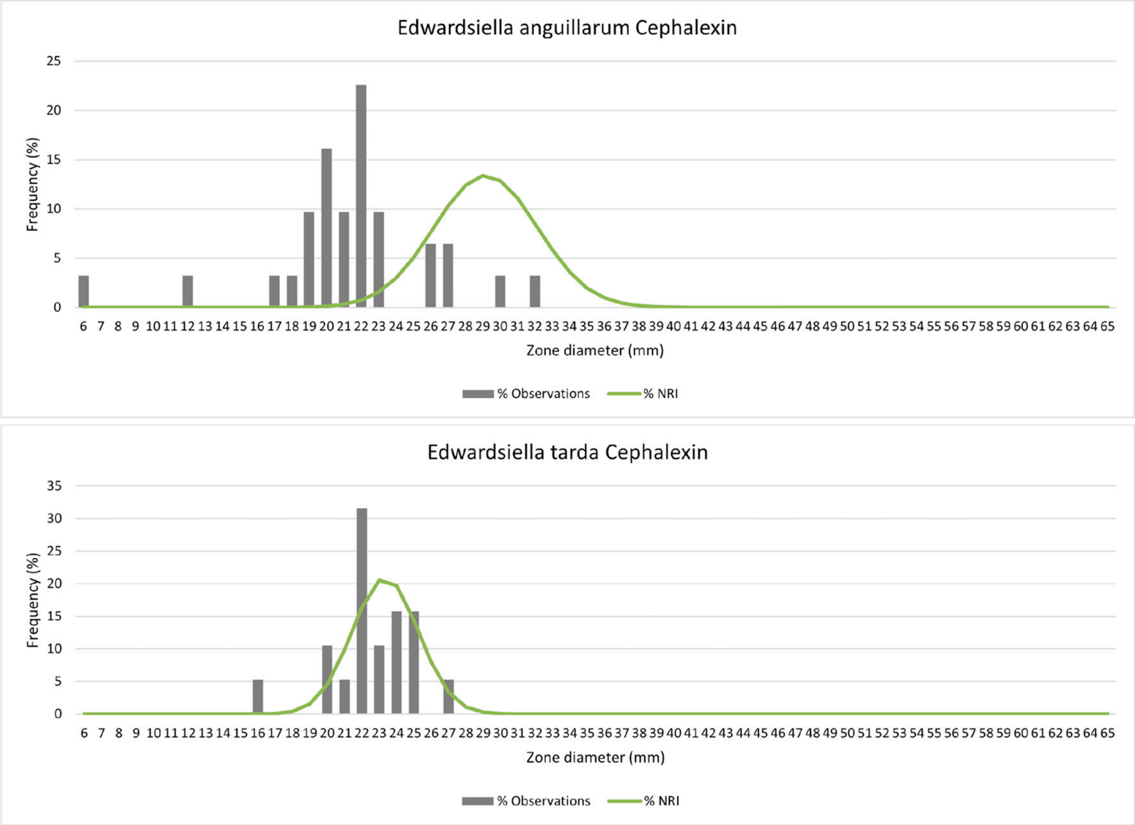

Supplementary Figure S14. NRI analysis of cephalexin inhibition zone diameters for *Edwardsiella anguillarum* and *Edwardsiella tarda*

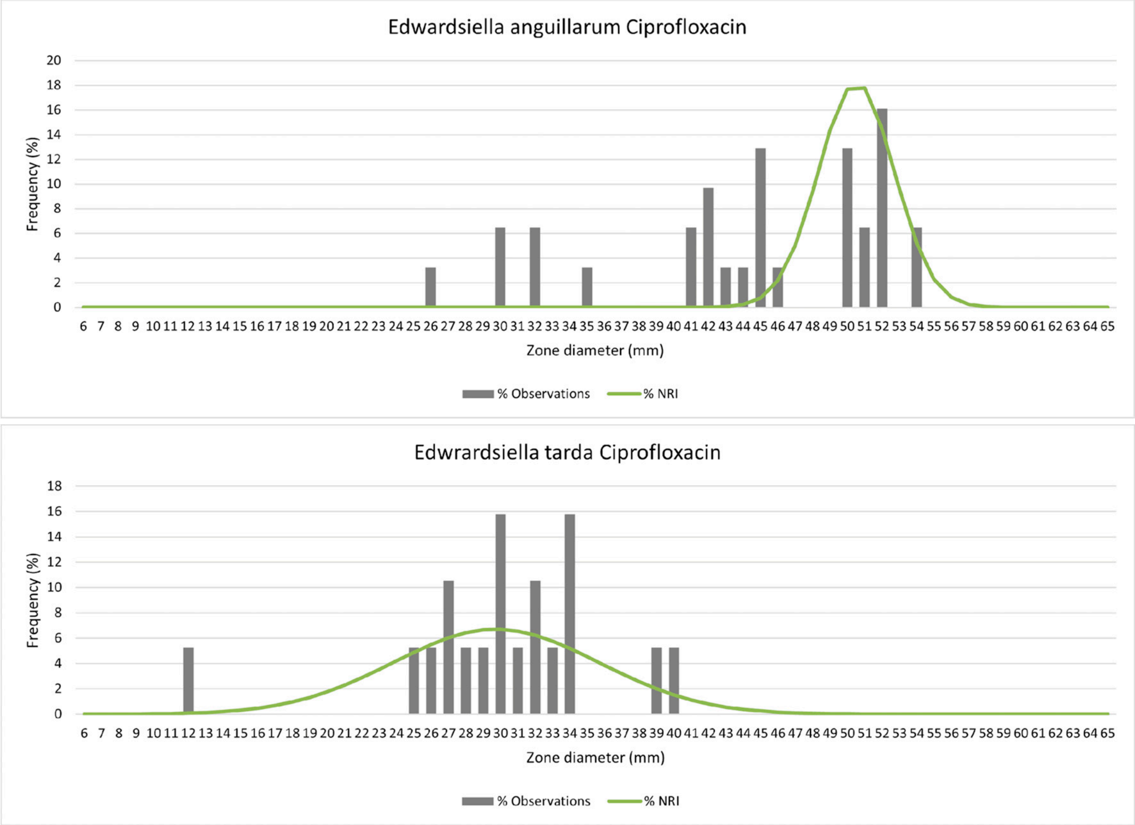

Supplementary Figure S15. NRI analysis of ciprofloxacin inhibition zone diameters for *Edwardsiella anguillarum* and *Edwardsiella tarda*

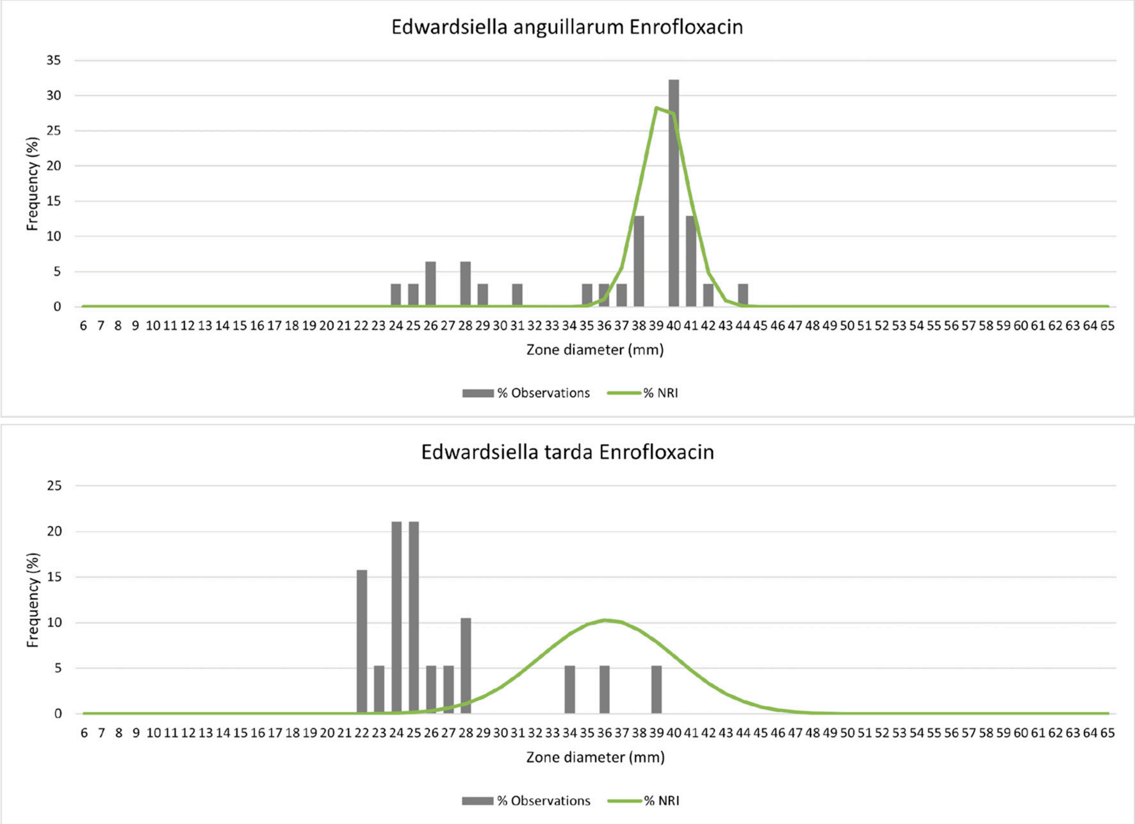

Supplementary Figure S16. NRI analysis of enrofloxacin inhibition zone diameters for *Edwardsiella anguillarum* and *Edwardsiella tarda*

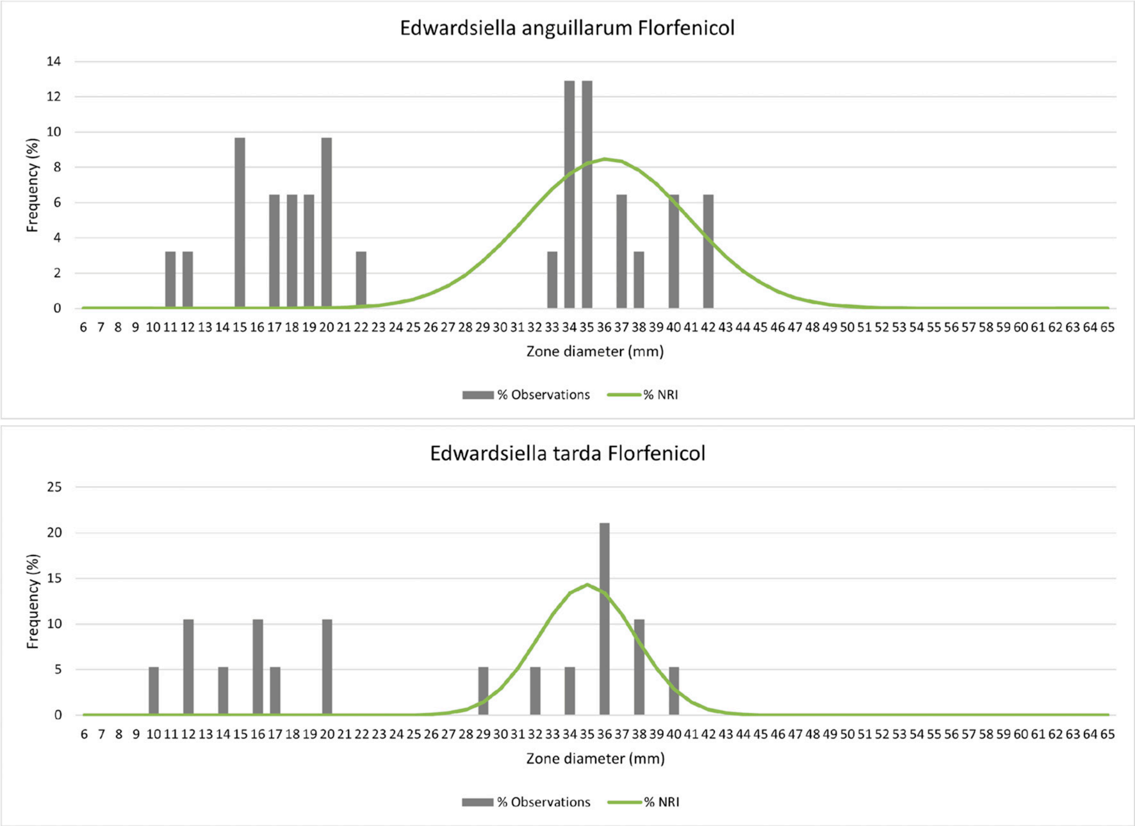

Supplementary Figure S17. NRI analysis of florfenicol inhibition zone diameters for *Edwardsiella anguillarum* and *Edwardsiella tarda*

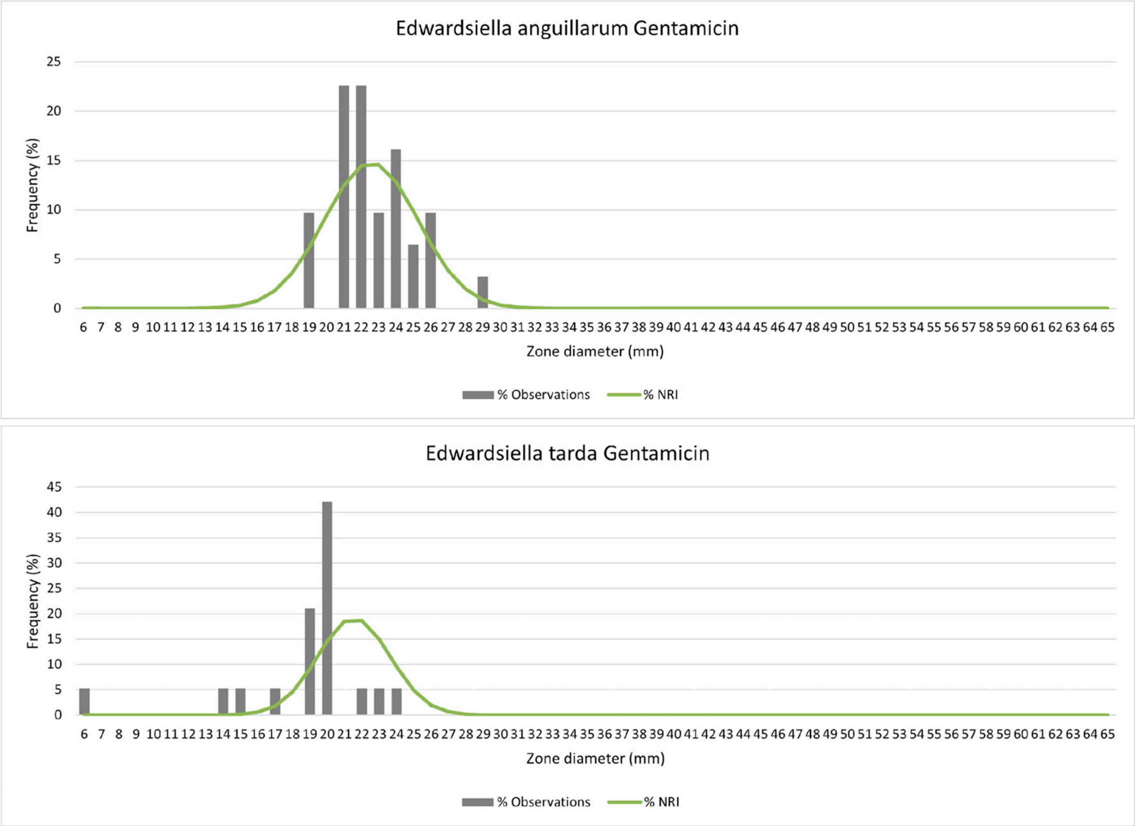

Supplementary Figure S18. NRI analysis of gentamicin inhibition zone diameters for *Edwardsiella anguillarum* and *Edwardsiella tarda*

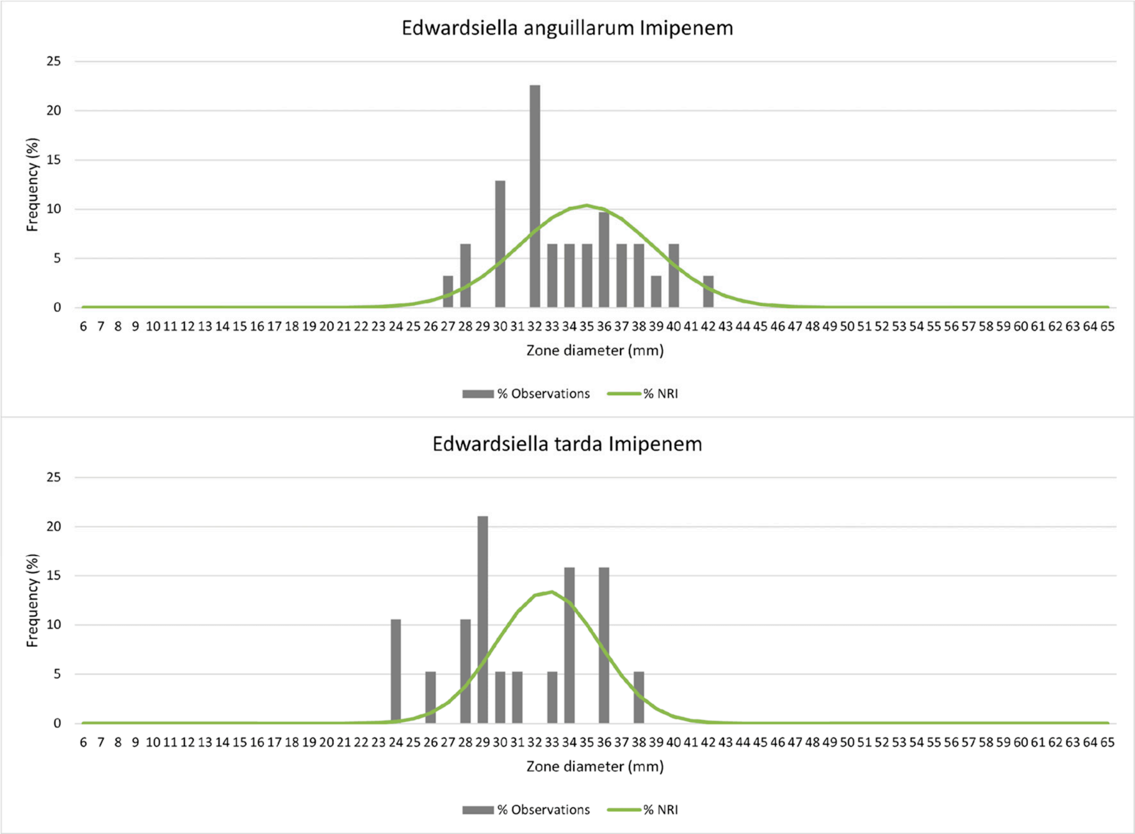

Supplementary Figure S19. NRI analysis of imipenem inhibition zone diameters for *Edwardsiella anguillarum* and *Edwardsiella tarda*

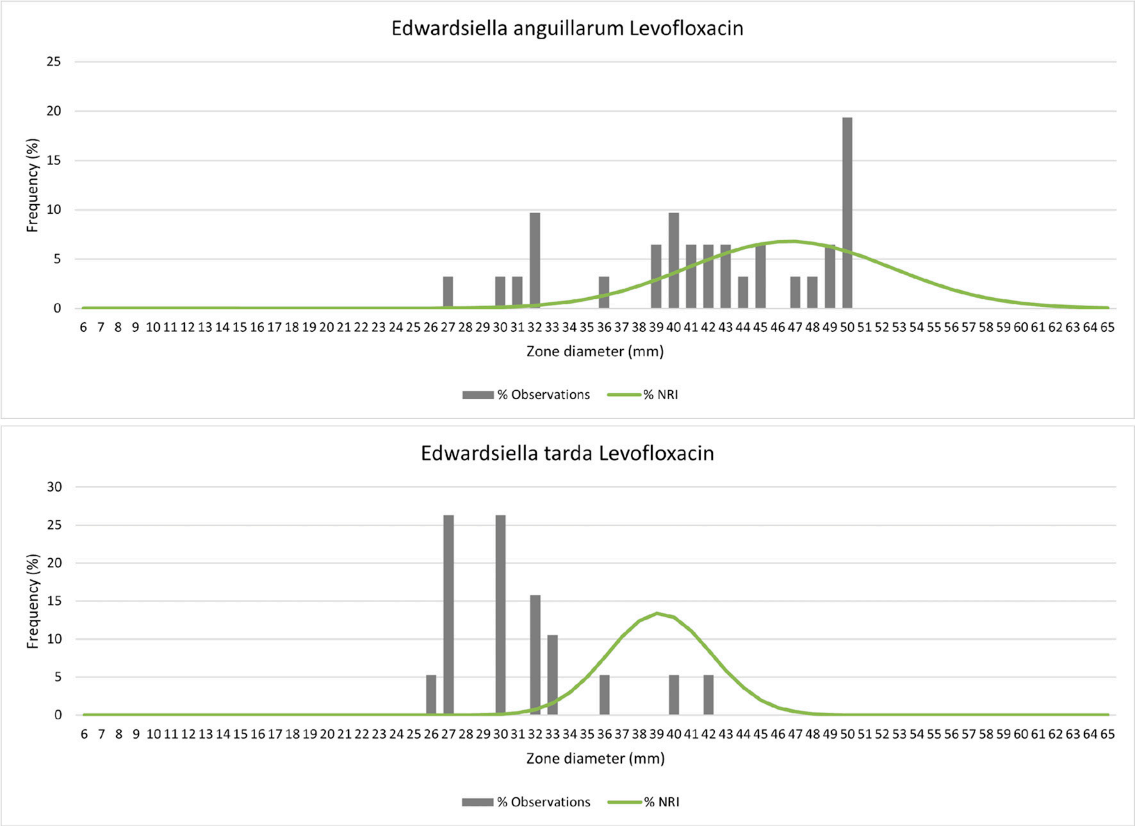

Supplementary Figure S20. NRI analysis of levofloxacin inhibition zone diameters for *Edwardsiella anguillarum* and *Edwardsiella tarda*

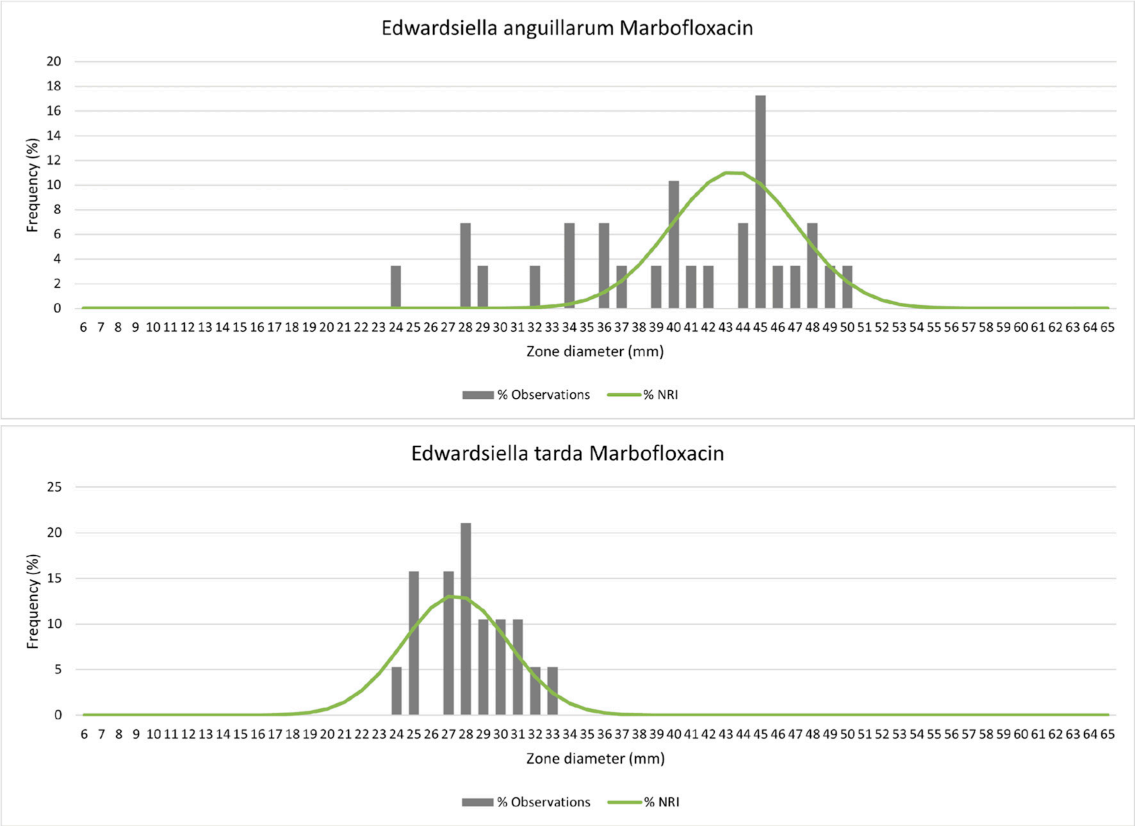

Supplementary Figure S21. NRI analysis of marbofloxacin inhibition zone diameters for *Edwardsiella anguillarum* and *Edwardsiella tarda*

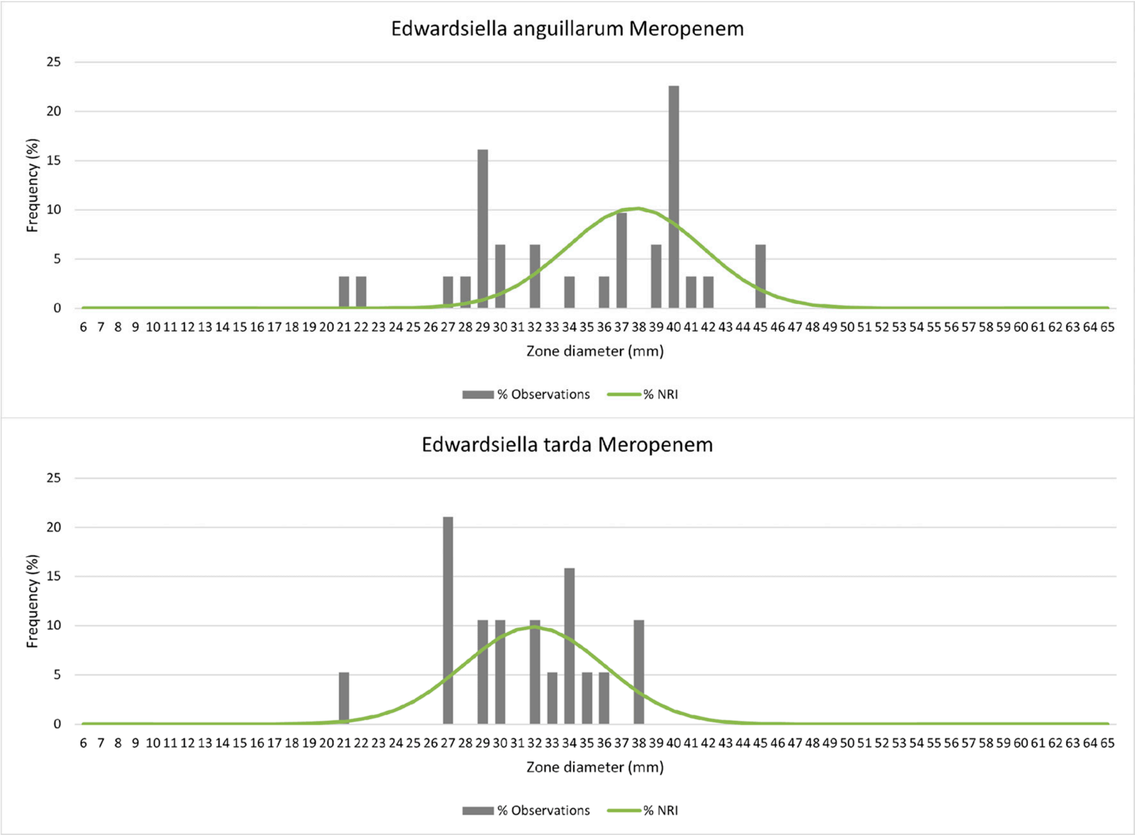

Supplementary Figure S22. NRI analysis of meropenem inhibition zone diameters for *Edwardsiella anguillarum* and *Edwardsiella tarda*

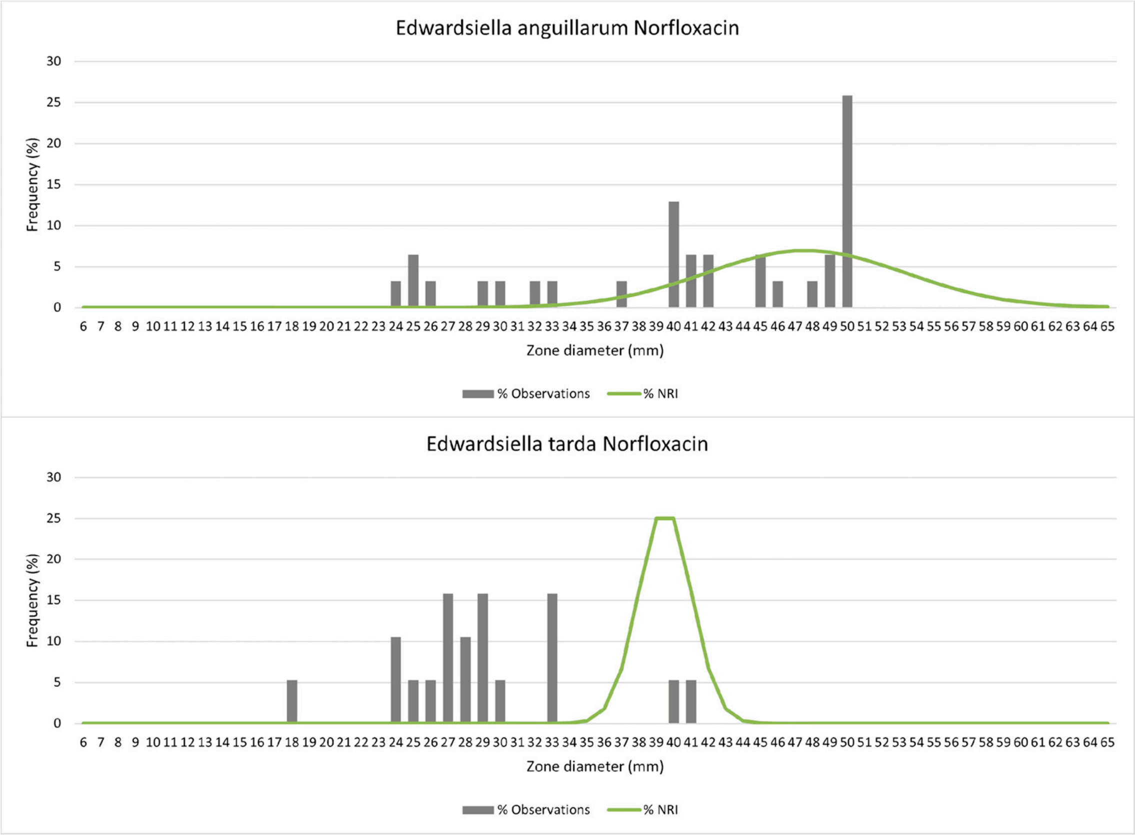

Supplementary Figure S23. NRI analysis of norfloxacin inhibition zone diameters for *Edwardsiella anguillarum* and *Edwardsiella tarda*

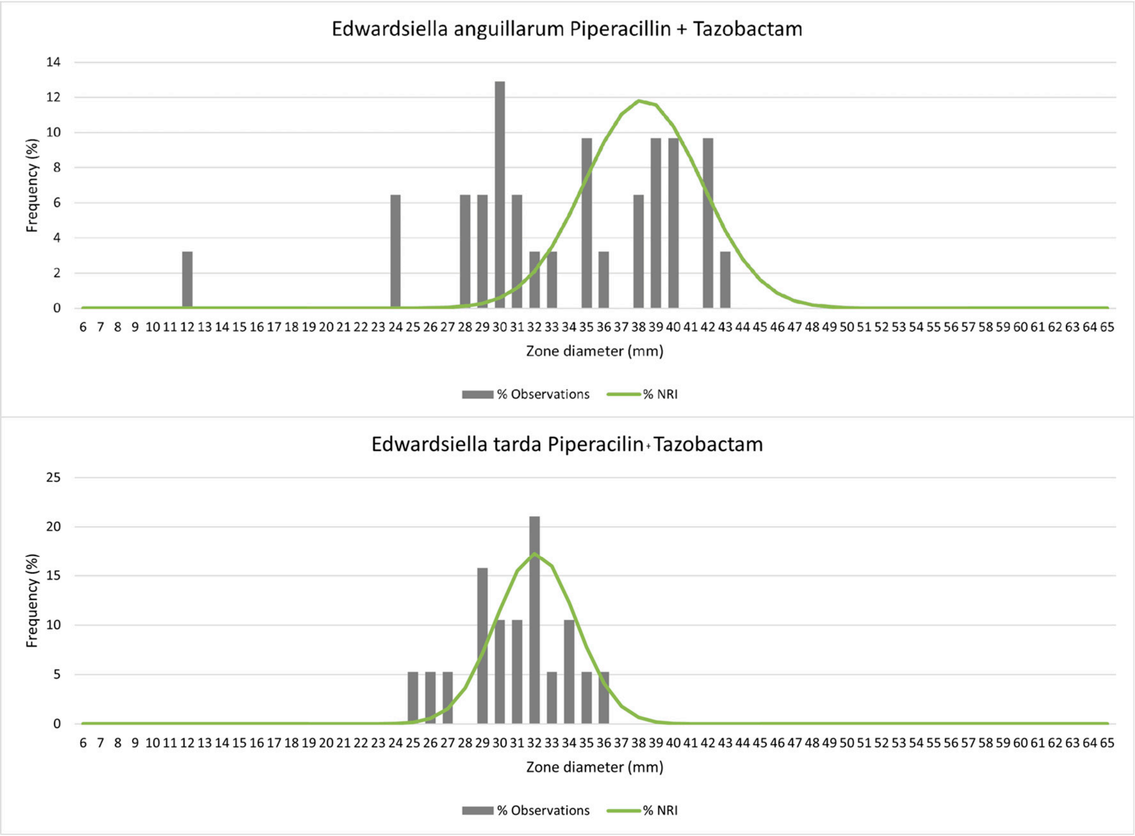

Supplementary Figure S24. NRI analysis of piperacillin + tazobactam inhibition zone diameters for *Edwardsiella anguillarum* and *Edwardsiella tarda*

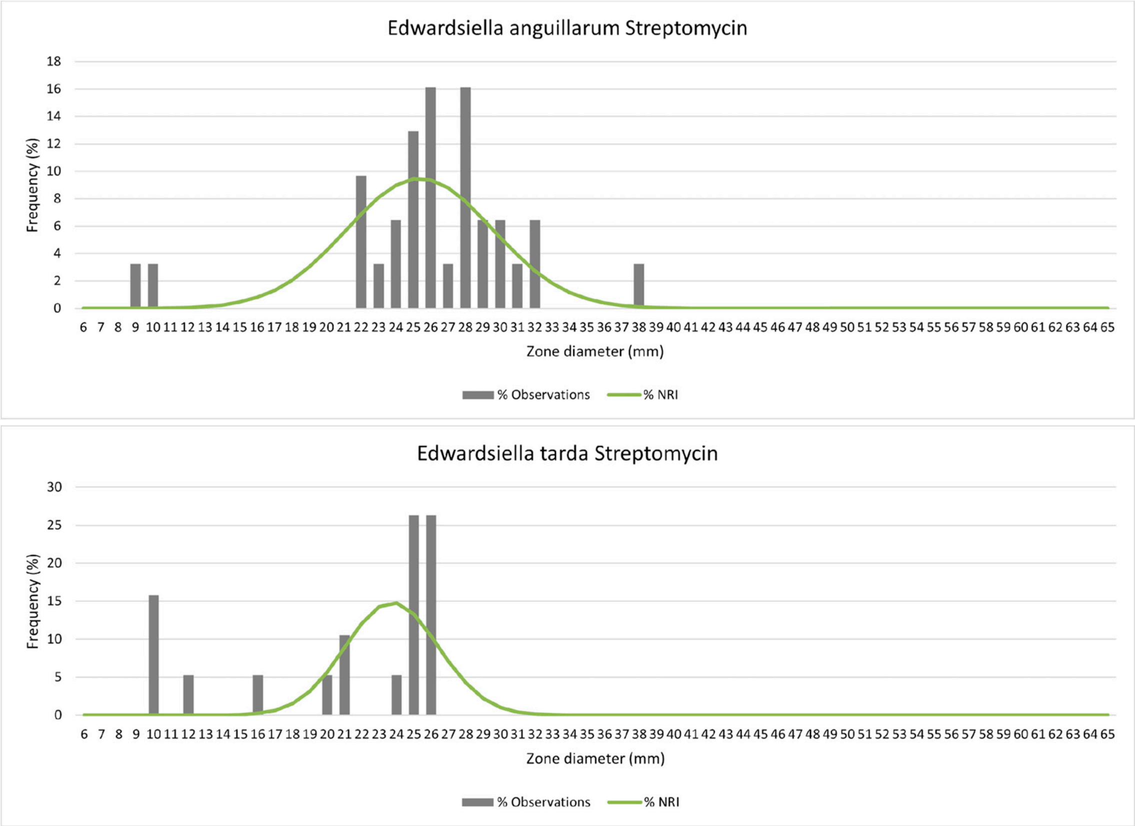

Supplementary Figure S25. NRI analysis of streptomycin inhibition zone diameters for *Edwardsiella anguillarum* and *Edwardsiella tarda*

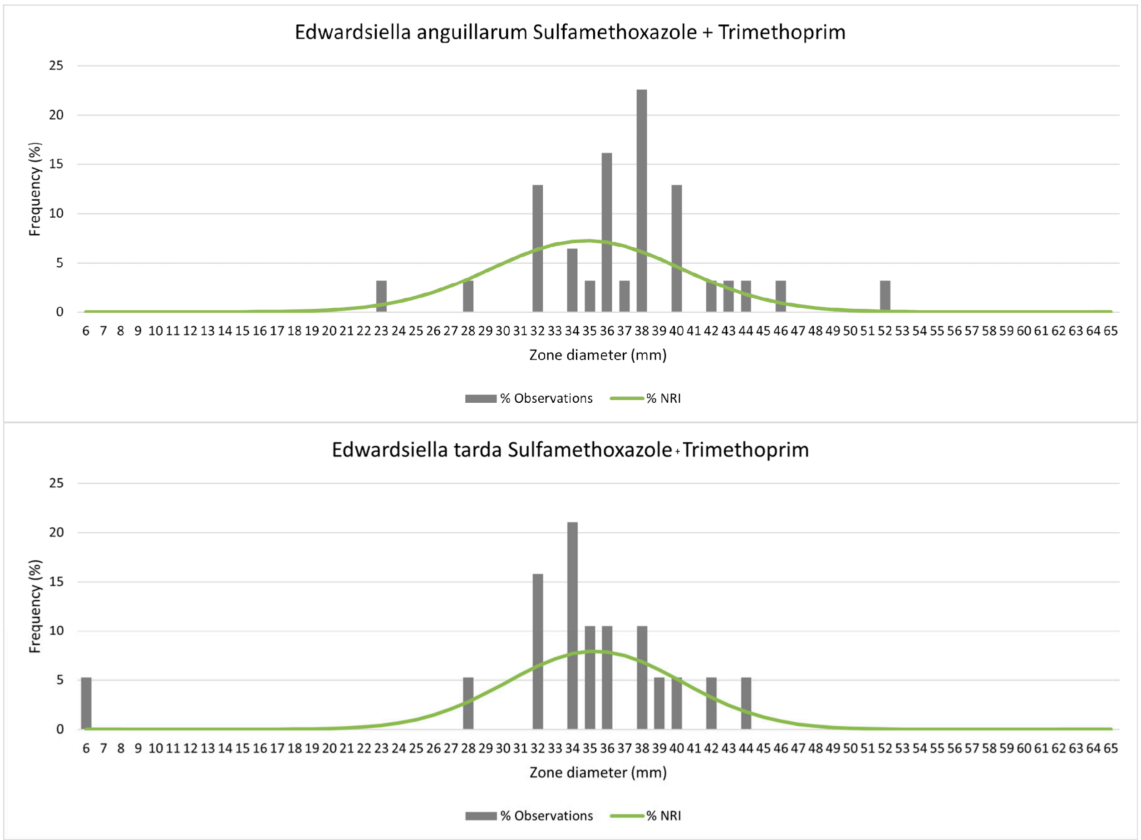

Supplementary Figure S26. NRI analysis of sulfazotrim inhibition zone diameters for *Edwardsiella anguillarum* and *Edwardsiella tarda*

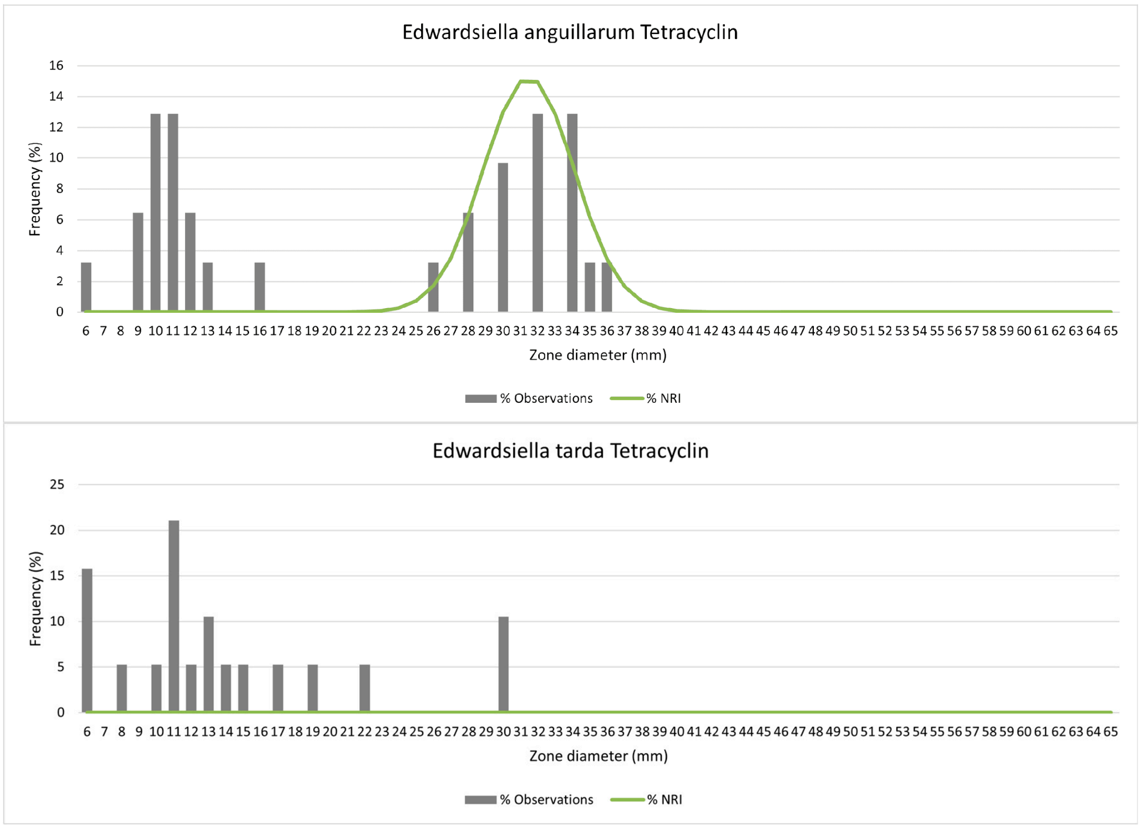

Supplementary Figure S27. NRI analysis of tetracyclin inhibition zone diameters for *Edwardsiella anguillarum* and *Edwardsiella tarda*

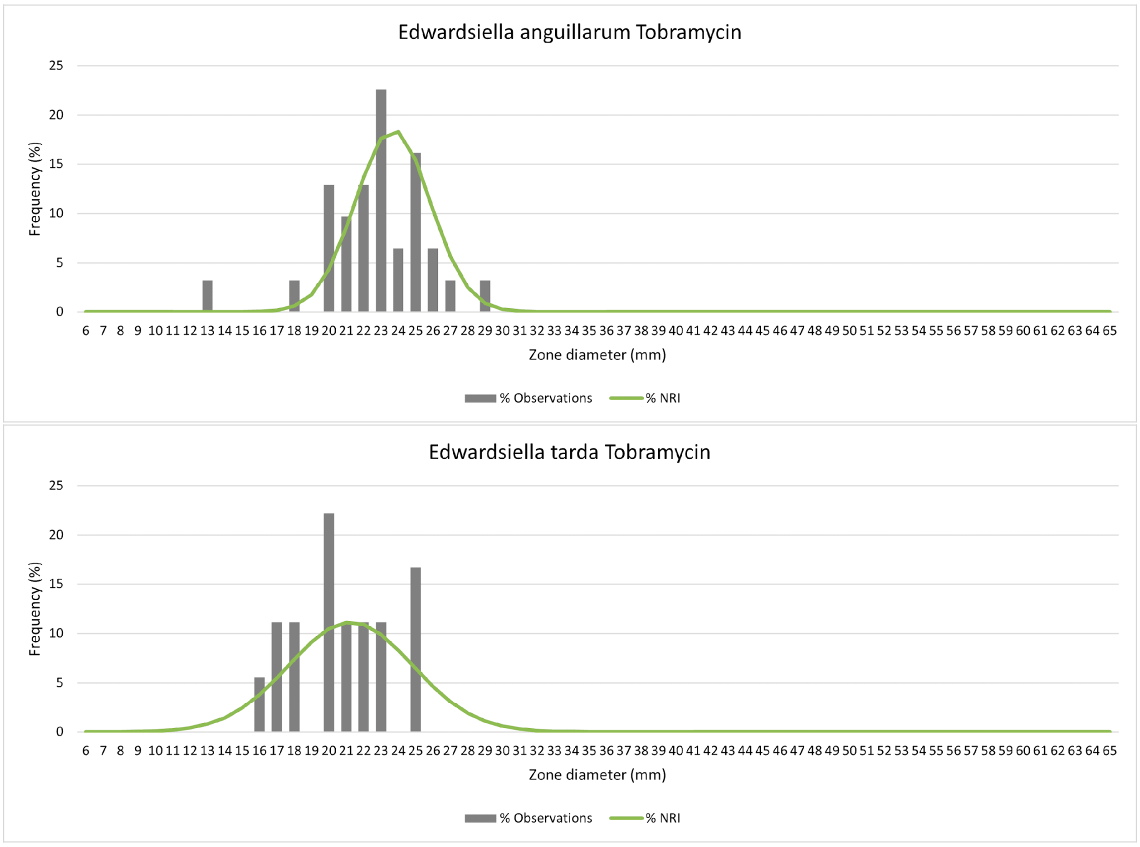

Supplementary Figure S28. NRI analysis of tobramycin inhibition zone diameters for *Edwardsiella anguillarum* and *Edwardsiella tarda*

Supplementary Table S1. Inhibition zone diameters (mm) for the quality control strain *Escherichia coli* ATCC 25922 used to validate antimicrobial susceptibility testing.

Escherichia coli ATCC25922			
Antimicrobials	Value (mm)	Antimicrobials	Value (mm)
Amikacin	21	Ciprofloxacin	30
Amoxicilin	19	Enrofloxacin	30
Amoxicilin + Clavulanic Acid	30	Florfenicol	30
Ampicilin	20	Gentamicin	23
Aztreonam	30	Imipenem	29
Cefazolin	22	Levofloxacin	30
Cefepime	30	Marbofloxacin	30
Cefotaxime	30	Meropenem	>30
Cefoxitin	26	Norfloxacin	30
Ceftazidime	27	Piperacilin + Tazobactam	25
Ceftiofur	30	Streptomicin	26
Ceftriaxone	30	Sulfazotrim	27
Cefuroxime	30	Tetracyclin	29
Cephalexin	20	Tobramicyn	17

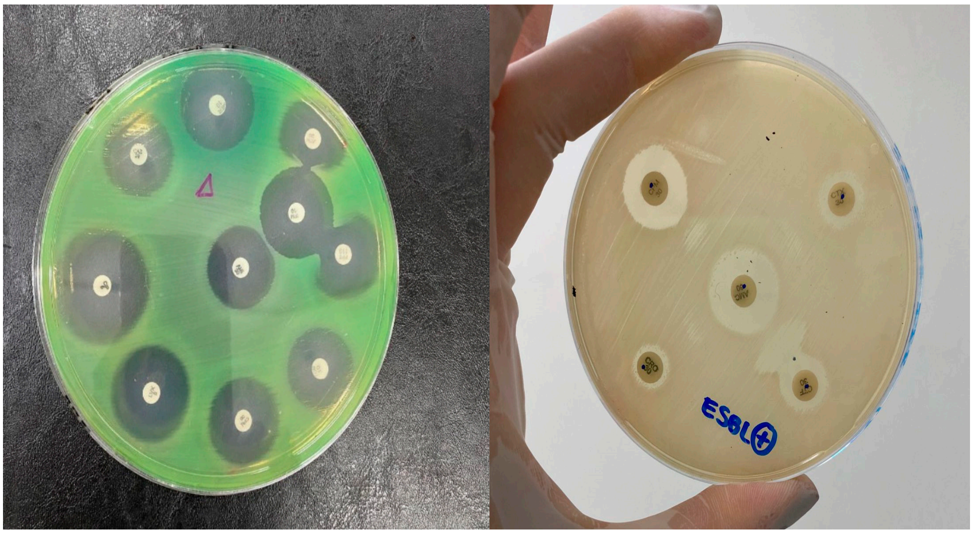

Supplementary Figure S29. Internal quality control plates confirming the performance of phenotypic assays for AmpC and ESBL β-lactamase detection.

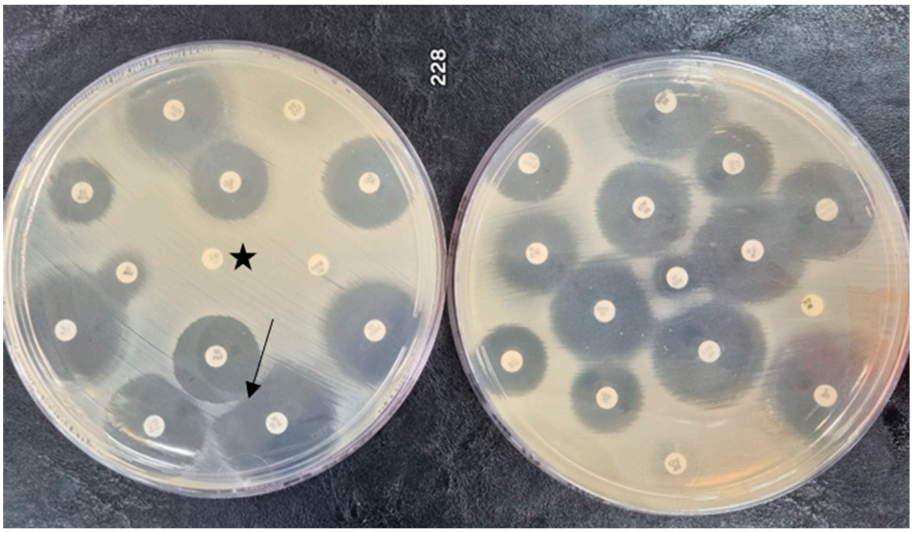

Supplementary Figure S30. Phenotypic detection of AmpC β-lactamase in the *Edwardsiella anguillarum* BEP228 isolate. Disk diffusion plates demonstrating the characteristic phenotypic profile of AmpC β-lactamase production by the Edwardsiella anguillarum BEP228 isolate. The star indicates the absence of an inhibition halo around the cephalothin (CFO) disk, a result suggestive of AmpC expression, since this enzyme hydrolyzes this antimicrobial. The arrow indicates the pattern of antagonism between aztreonam (ATM) and imipenem (IPM), observed by the distortion of the halo, reinforcing the phenotypic suspicion of AmpC production.
